# Evolutionary and Phenotypic Characterization of Two Spike Mutations in European Lineage 20E of SARS-CoV-2

**DOI:** 10.1128/mBio.02315-21

**Published:** 2021-11-16

**Authors:** Paula Ruiz-Rodriguez, Clara Francés-Gómez, Álvaro Chiner-Oms, Mariana G. López, Santiago Jiménez-Serrano, Irving Cancino-Muñoz, Paula Ruiz-Hueso, Manuela Torres-Puente, Maria Alma Bracho, Giuseppe D’Auria, Llúcia Martinez-Priego, Manuel Guerreiro, Marta Montero-Alonso, María Dolores Gómez, José Luis Piñana, SeqCOVID-SPAIN Consortium, Fernando González-Candelas, Iñaki Comas, Alberto Marina, Ron Geller, Mireia Coscolla

**Affiliations:** a I^2^SysBio, University of Valencia -CSIC, FISABIO Joint Research Unit Infection and Public Health, Valencia, Spain; b Instituto de Biomedicina de Valencia, Consejo Superior de Investigaciones Científicas, Valencia, Spain; c Sequencing and Bioinformatics Service of Valencian Region Foundation for the Promotion of Health and Biomedical Research, Valencia, Spain; d CIBER in Epidemiology and Public Health, Madrid, Spain; e Hematology Department, Hospital Universitari i Politècnic la Fe, Valencia, Spain; f Instituto de Investigación Sanitaria La Fe, Valencia, Spain; g Infectious Diseases Unit, Hospital Universitari i Politècnic la Fe, Valencia, Spain; h Microbiology Department, Hospital Universitari i Politècnic La Fe, Valencia, Spain; i Hematology Service, Hospital Clínico Universitario, Institute for Research INCLIVA, Valencia, Spain; j CIBER in Rare Diseases, Madrid, Spain; Institut Pasteur

**Keywords:** SARS-CoV-2, spike, HR2, variants, homoplasy, antibody escape, adaptive mutations

## Abstract

We have detected two mutations in the spike protein of severe acute respiratory syndrome coronavirus 2 (SARS-CoV-2) at amino acid positions 1163 and 1167 that appeared independently in multiple transmission clusters and different genetic backgrounds. Furthermore, both mutations appeared together in a cluster of 1,627 sequences belonging to clade 20E. This cluster is characterized by 12 additional single nucleotide polymorphisms but no deletions. The available structural information on the S protein in the pre- and postfusion conformations predicts that both mutations confer rigidity, which could potentially decrease viral fitness. Accordingly, we observed reduced infectivity of this spike genotype relative to the ancestral 20E sequence *in vitro*, and the levels of viral RNA in nasopharyngeal swabs were not significantly higher. Furthermore, the mutations did not impact thermal stability or antibody neutralization by sera from vaccinated individuals but moderately reduce neutralization by convalescent-phase sera from the early stages of the pandemic. Despite multiple successful appearances of the two spike mutations during the first year of SARS-CoV-2 evolution, the genotype with both mutations was displaced upon the expansion of the 20I (Alpha) variant. The midterm fate of the genotype investigated was consistent with the lack of advantage observed in the clinical and experimental data.

## INTRODUCTION

Genomic surveillance of viral mutations is the first step in detecting viral changes that could impact public health by interfering with diagnostics, modifying pathogenicity, or altering susceptibility to existing immunity or treatments. In many countries, the challenge of detecting new mutations of interest in severe acute respiratory syndrome coronavirus 2 (SARS-CoV-2) involves sequencing representative genomes from circulating viruses, sharing sequence information on public databases (e.g., GISAID [1]), and analyzing them in real time using platforms such as Nextstrain (2). While mutations appear randomly, their fate in the population depends on a combination of the conferred fitness advantage and stochastic and demographic processes. A first step in assessing the potential public health impact of newly observed mutations is to determine whether their increase in frequency is due to chance or adaptation. If they are found to be adaptive, it is important to evaluate whether their adaptation is linked to an improved ability to replicate, colonize, transmit, or evade antiviral hosts defenses (3). An important challenge in the field is to decipher which of all the variants that appear should be monitored to implement measures that mitigate their risk to public health.

Mutations in SARS-CoV-2 have been reported since the early stages of the coronavirus disease 2019 (COVID-19) epidemic (4–6). The most common mutations described are single nucleotide polymorphisms (SNPs) and small deletions (7–9). Genomic surveillance of mutations has been mostly focused on the spike (S) protein because of its key role in viral entry and immunity (10), as well as the fact that this protein constitutes the basis of numerous SARS-CoV-2 vaccines (11). S is a homotrimeric protein whose heavily glycosylated ectodomain protrudes from the viral membrane, showing a bat-like shape with an N-terminal globular head connected to the membrane by an elongated stalk (12). The S protein is proteolytically processed by the cellular furin protease into the S1 and S2 subunits (13, 14). Additional proteolytic cleavage occurs following the binding of the S protein to host receptors, facilitating S1 subunit release. The C-terminal S2 subunit remains trimeric in the viral membrane but undergoes conformational changes that promote fusion with the host cell (15).

The first mutation identified as potentially concerning was a change from aspartic acid to glycine in the S1 subunit of the S protein at position 614 (S:D614G). S:D614G emerged early in the epidemic, becoming predominant in most countries within 2 months, and completely dominated the epidemic by August 2020 (16). As with any mutant, the initial spread of this mutation could have resulted from stochastic events, the dynamics of the epidemic, or an intrinsically higher viral fitness. More than 6 months after the initial report of this mutation, several studies reported evidence in favor of higher transmission efficacy in animal models and human populations (4, 17–19). S:D614G replicates better in some cell culture and animal models (17, 18, 20) and is associated with higher viral loads in infected individuals (16); importantly, however, it does not impact diagnostics or vaccine efficacy.

Following the first wave of the pandemic, additional variants have been reported from many countries. Among the first of these changes associated with variants in the spike protein was the amino acid replacement S:A222V, located at the N-terminal domain (NTD) of the S1 subunit, which occurred in the background of S:D614G. The variant containing this change, termed 20E, was first sequenced in Spain and expanded throughout Europe (5). Other variants have been reported since, including the so-called cluster 5 variant, which harbors a combination of 3 SNPs and a single deletion related to mink farms in Denmark (21). One of the SNPs is in the S protein of this variant, S:Y453F; it occurs in the receptor-binding domain (RBD) and may increase binding to cell receptors in mink (22). By late 2020 or early 2021, three variants of concern (VOC) were described, all of which share the S:N501Y amino acid replacement in the RBD of the S protein: Alpha (also called 20I/501Y.V1 or lineage B.1.1.7) was originally described in the United Kingdom (9), Beta (20H/501Y.V2; B.1.351) in South Africa, and Gamma (20J/501Y.V3; P.1) in Brazil. Recently, in March 2021, a new VOC known as Delta (21A/478K.V1, B.1.617.2) emerged in India. The Delta variant does not contain S:501Y (23) and is displacing the predominant variant, Alpha (24). These variants are of particular concern because of their rapid spread, likely due to increased transmissibility (25–27). Reduction in neutralization has been found in different amino acids of the spike protein. VOC with the amino acid replacement S:N501Y (Alpha, Beta, and Gamma) exhibit the highest impact on immune evasion, followed by lineages harboring S:L452R that include the Delta variant (B.1.617.2) (28). While the effect of these variants on the immune response in convalescent and vaccinated individuals is still unclear, current data do not provide evidence of immune escape or compromising vaccine efficacy (29). Nevertheless, new mutations could emerge that hamper efforts to control the epidemic at regional or global scales by increasing transmissibility and/or reducing vaccine efficacy in the future.

The dominance of a lineage in a geographical region is sometimes determined by the number of introductions and mobility among regions (30) rather than by a change in a biological trait that confers a selective advantage (5). Nearly all VOC thus far have spread outside the country where they were initially identified and are estimated to spread faster than other cocirculating genotypes, becoming dominant for a period (25, 31, 32) and eventually being replaced locally by other variants (24). The current work describes the workflow for investigating the risk of emerging mutations in the spike protein of SARS-CoV-2, starting from genomic epidemiology and leading up to a biological and immunological characterization of SARS-CoV-2 mutations in terms of viral infectivity, virion stability, and neutralization by sera from convalescent and vaccinated individuals.

## RESULTS

### Multiple and independent mutations in amino acid positions 1163 and 1167 of the spike protein.

SARS-CoV-2 genetic variation has been monitored by the Spanish sequencing consortium SeqCOVID to follow the expansion of mutations that could potentially result in a change of the biological properties of the virus. We focused on mutations in the S protein because of its relevance for infection and immunity (10). We detected two mutations in the S gene: G25049T (S:D1163Y) and G25062T (S:G1167V), which appeared in Spain as early as March and April 2020, respectively (see Fig. S1 in the supplemental material). These mutations continued arising independently of each other and, by the end of June, when the predominant circulating genotypes from the first wave in Spain had already been replaced by other variants (30), were also observed together (Fig. S1). Both positions have mutated multiple times independently and to different amino acids at a lower frequency. On the one hand, S:D1163 appears to have mutated at least 99 times (S:D1163Y, 84; S:D1163V, 4; S:D1163G, 3; S:D1163A, 2; S:D1163E, 2; S:D1163H, 2; S:D1163N, 1; and S:D1163H/Y, 1) in 47 lineages according to the pangolin scheme (33). On the other hand, S:G1167 appears to have mutated at least 54 times (S:G1167V, 39; S:G1167D, 4; S:G1167C, 3; S:G1167R, 3; S:G1167S, 3; S:G1167F, 1; and S:G1167A, 1) in 20 PANGO lineages, including B.1 (Fig. S2e) and its derivatives B.26, B.40 (Fig. S2c), and D.2 (Fig. S2f).

10.1128/mBio.02315-21.5 FIG S1 Temporal distribution of mutated samples colored by region. (a) Distribution of amino acid replacement S:D1163Y over the pandemic (*n* = 1,874). (b) Distribution of amino acid replacement S:G1167V over time (*n* = 1,708). Download FIG S1, PDF file, 0.2 MB. Copyright © 2021 Ruiz-Rodriguez et al. 2021 Ruiz-Rodriguez et al. https://creativecommons.org/licenses/by/4.0/ This content is distributed under the terms of the Creative Commons Attribution 4.0 International license.

10.1128/mBio.02315-21.6 FIG S2 Maximum-likelihood phylogenies for different PANGO lineages. (a) Complete phylogeny colored by the PANGO lineages. (b) Phylogeny of lineage B.53. The circle represents sequences with D1163 amino acid replacement. (c) Phylogeny of lineage B.40. The inner circle represents sequences with S:D1163 amino acid replacements, and the outer circle represents sequences with S:G1167 amino acid replacements. (d) Phylogeny of lineage A. The circle represents sequences with S:D1163 amino acid replacements. (e) Phylogeny of B.1 and derived lineages. The inner circle represents sequences with S:D1163 amino acid replacements, and the outer circle represents sequences with S:G1167 amino acid replacements. (f) Phylogeny of B.1.1 and derivative D.1 lineages. The inner circle represents sequences with S:D1163 amino acid replacements, and the outer circle represents sequences with S:G1167 amino acid replacements. (g) Phylogeny of 20E and 1163.7. The inner circle represents sequences with S:D1163 amino acid replacements, and the external circle represents sequences with S:G1167 amino acid replacements. Each scale bar indicates the number of nucleotide substitutions per site. The legend for positions S:1163 and S:1167 is common to all panels. Download FIG S2, PDF file, 2.4 MB. Copyright © 2021 Ruiz-Rodriguez et al. 2021 Ruiz-Rodriguez et al. https://creativecommons.org/licenses/by/4.0/ This content is distributed under the terms of the Creative Commons Attribution 4.0 International license.

### Clusters of transmission with amino acid changes in positions 1163 and 1167 of spike.

**FIG 1:**
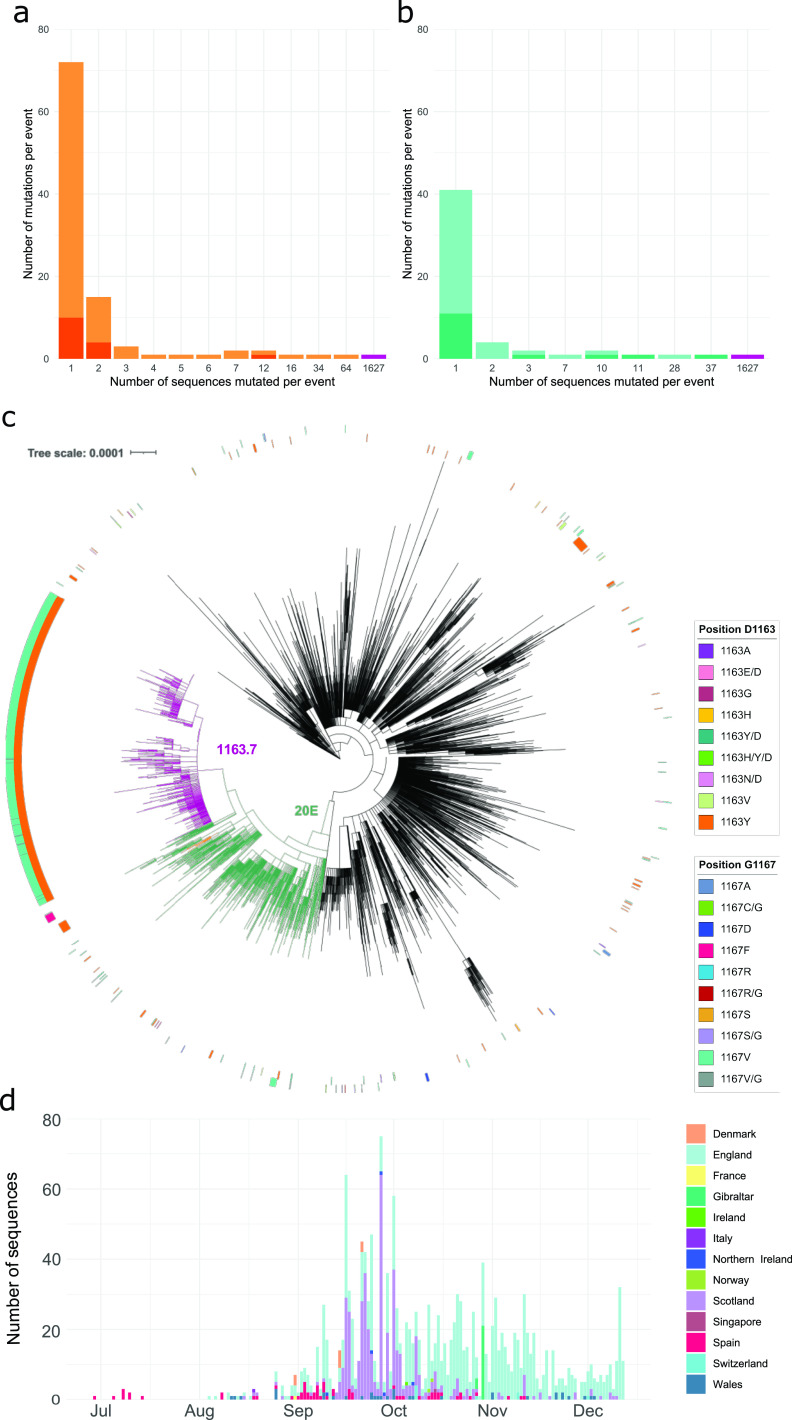
Sequences mutated at positions 1163 and 1167 of the S protein. (a) The number of mutation events for amino acid replacement S:D1163Y (light orange) or another S:D1163 amino acid replacements (dark orange). (b) The number of mutation events for amino acid replacement S:G1167V (light turquoise) or another S:G1167 amino acid replacements (dark turquoise). Bars in magenta indicate the appearance of both the S:D1163Y and S:G1167V amino acid replacements in the same sequences. (c) Maximum-likelihood phylogeny of 10,450 SARS-CoV-2 genomes. The inner ring represents sequences with amino acid changes in position D1163 of the S protein. The outer ring represents sequences with amino acid changes in position G1167 of the S protein. Branches are colored in magenta for 1163.7, green for clade 20E, and orange for cluster 1163.654. The scale bar indicates the number of nucleotide substitutions per site. (d) Temporal distribution and frequency of sequences with variant 1163.7 colored by geographical origin.

The majority of mutated sequences in position 1163 and 1167 in the S protein (94.43%) were found in transmission clusters (see Materials and Methods for the definition of clusters) (Fig. 1a and b), with a small minority not belonging to a transmission cluster due to either incomplete sampling or failure to spread. While different amino acids changes have been detected at both positions, only one change at each position appeared in most clusters: S:D1163Y in 83.33% and S:G1167V in 69.23% of clusters. S:D1163Y appeared in 22 transmission clusters (Fig. 1a) and S:G1167V in 8 clusters (Fig. 1b). Interestingly, the largest cluster included both the S:D1163Y and S:G1167V amino acid replacements together and was detected initially in 65 sequences from Spain until December 2020, representing 1.17% of the Spanish sequences and 1,627 sequences in total, representing 0.60% of sequences globally (Fig. 1c and d). The 1,627 sequences form a monophyletic cluster within lineage 20E (also described as 20E.EU1 [5] and B.1.177 [33]), which we designate cluster 1163.7. Cluster 1163.7 is characterized by nine nonsynonymous and six synonymous mutations with respect to the reference sequence from Wuhan (Table S2) but lacks any shared deletions. The amino acid changes A222V, D614G, D1163Y, and G1167V were found in the S protein, A220V and P365S were found in the N protein, V30L was found in ORF10, L67F was found in ORF14, and P4715L was found in ORF1ab (Fig. S3 and Table S2). Synonymous mutations were also observed in the ORF1ab, N, and M genes (Fig. S3 and Table S2).

10.1128/mBio.02315-21.3 TABLE S2 Defining SNPs for 1163.7, 1163.7.V2, and 1163.654. Download Table S2, XLSX file, 0.01 MB. Copyright © 2021 Ruiz-Rodriguez et al. 2021 Ruiz-Rodriguez et al. https://creativecommons.org/licenses/by/4.0/ This content is distributed under the terms of the Creative Commons Attribution 4.0 International license.

10.1128/mBio.02315-21.7 FIG S3 Whole-genome mutated positions in genotypes with changes D1163Y and 1167V of the S protein. Cluster 1163.7 is in magenta, and cluster 1163.654 is in orange. Other less frequent genotypes (found in at least 20 sequences) that include changes in S:1163 and/or S:1167 are in turquoise. Cluster 1163.654 is in navy blue. Line width is proportional to the frequency of the genotype. Sites S:1163 and S:1167 are indicated by magenta stars, and position S:E484K, whose mutations are associated with antigenicity changes, is indicated by a navy blue star. Download FIG S3, PDF file, 0.2 MB. Copyright © 2021 Ruiz-Rodriguez et al. 2021 Ruiz-Rodriguez et al. https://creativecommons.org/licenses/by/4.0/ This content is distributed under the terms of the Creative Commons Attribution 4.0 International license.

Within 20E, the second largest cluster including any of these mutations was observed in 34 sequences with E654Q and D1163Y in S protein plus an additional 7 nonsynonymous and 6 synonymous mutations (Table S2 and Fig. S3). We designate this second cluster, which is also embedded within lineage 20E, cluster 1163.654 (Fig. 1c and Fig. S3). Cluster 1163.654 appeared first in Ireland on 23 July 2020 and subsequently appeared in Spain and England. However, cluster 1163.654 was no longer detected after 3 months.

Because of the risk posed by VOC (9, 32, 35), we examined whether mutations involving 1163 and 1167 of the S protein were observed in VOC until July 2021. We detected one of the mutations in 170 Delta, 676 Alpha, 147 Beta, and 153 Gamma sequences. Interestingly, S:D1163Y and S:G1167V were observed together in only one individual with 20I, the Alpha variant, although both positions showed polymorphism within the individual (relative frequency of 27% and 17% of the reads with S:D1163Y and S:G1167V, respectively).

### Evolution of 1163.7.

**FIG 2:**
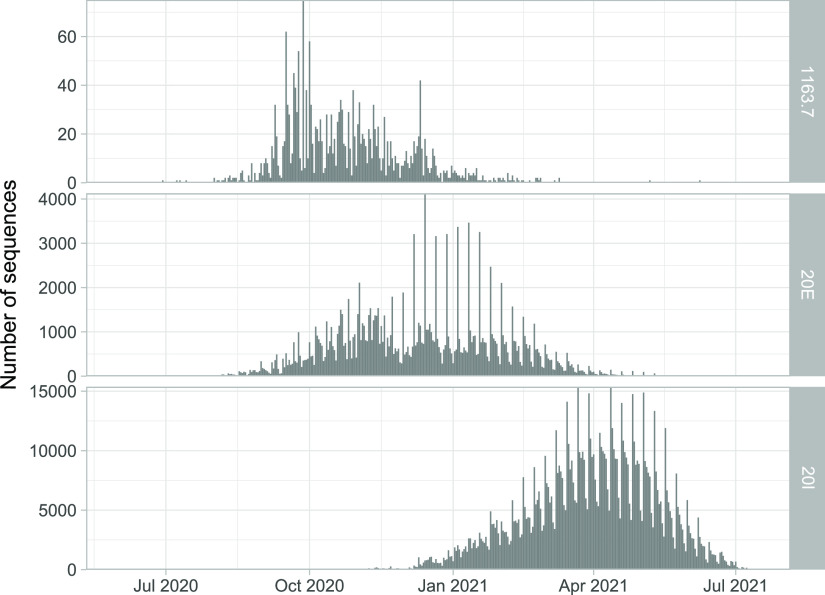
Temporal distribution of sequences in GISAID per variant. Number of sequences classified as cluster 1163.7 (*n* = 2,106), 20E (*n* = 159,450), and 20I (*n* = 979,013) by date from June 2020 until the beginning of July 2021.

We explored the emergence and evolution of 1163.7, the largest and most successful cluster involving amino acid changes in positions 1163 and 1167 of the S protein. 1163.7 appeared in Spain in June 2020 in sequences from the Basque Country (Fig. 1d and Video S1) and subsequently appeared in individuals from other countries, accounting for a total of 1,627 sequences in GISAID (0.60% of 270,869 analyzed sequences by 23 December 2020) (Fig. 1d and Video S1). The majority of the 1163.7 sequences were obtained from the United Kingdom, including England (*n* = 1,058), Scotland (*n* = 419), Wales (*n* = 34), and Northern Ireland (5) but were also observed in Gibraltar (24 sequences), indicating successful migration and transmission (Video S1). Although 1163.7 is not well represented in sequences from other countries, it has been found in multiple sequences from Denmark (*n* = 9), Switzerland (*n* = 8), and Norway (*n* = 2) and single sequences from Italy, France, Singapore, and Ireland. By the end of 2020, 1163.7 was still circulating in Europe (Fig. 1d and Video S1), and it was represented by 1,923 sequences in GISAID by the end of February 2021 (0.33% of submitted sequences). After this time point, when VOC were increasing in frequency, 1163.7 ceased to be detected (Fig. 2), being replaced by the Alpha variant similarly to other variants in Europe, such as 20E.

Within 1163.7, we detected additional SNPs in individual sequences or small groups of sequences. One of these changes is E484K in the RDB of the S protein, a mutation present in three VOC (Alpha, Beta, and Gamma) that is implicated in increased ACE2 binding (36) and reduced neutralization by antibodies (37). In addition, we found another change associated with evasion of antibody immunity: a deletion of positions 141 to 144 in the S protein, which partially overlaps a smaller deletion at 144 reported in VOC Alpha (38). This subcluster included five sequences during January of 2021 from England and Wales (Table S2). The five sequences formed a monophyletic group embedded in 1163.7 (Fig. S5), identified as cluster 1163.7.V2, which displays other nonsynonymous and synonymous mutations (Table S2), and only two sites are polymorphic within 1163.7.V2.

10.1128/mBio.02315-21.9 FIG S5 Maximum-likelihood phylogeny of 3,266 SARS-CoV-2 genomes representing 20E clade rooted with the reference sequence. Sequences from 1163.7 are in magenta, sequences not identified as 1163.7 are in green, and cluster 1163.7.V2 (S protein amino acid replacements: A222V, D614G, E484K, D1163Y, and 141-144Del) is in blue. The scale bar indicates the number of nucleotide substitutions per site. The biggest clades are collapsed and represented with isosceles triangles. Download FIG S5, PDF file, 0.05 MB. Copyright © 2021 Ruiz-Rodriguez et al. 2021 Ruiz-Rodriguez et al. https://creativecommons.org/licenses/by/4.0/ This content is distributed under the terms of the Creative Commons Attribution 4.0 International license.

### Positions 1163 and 1167 of the S protein are located in the heptad repeat 2 motif.

**FIG 3:**
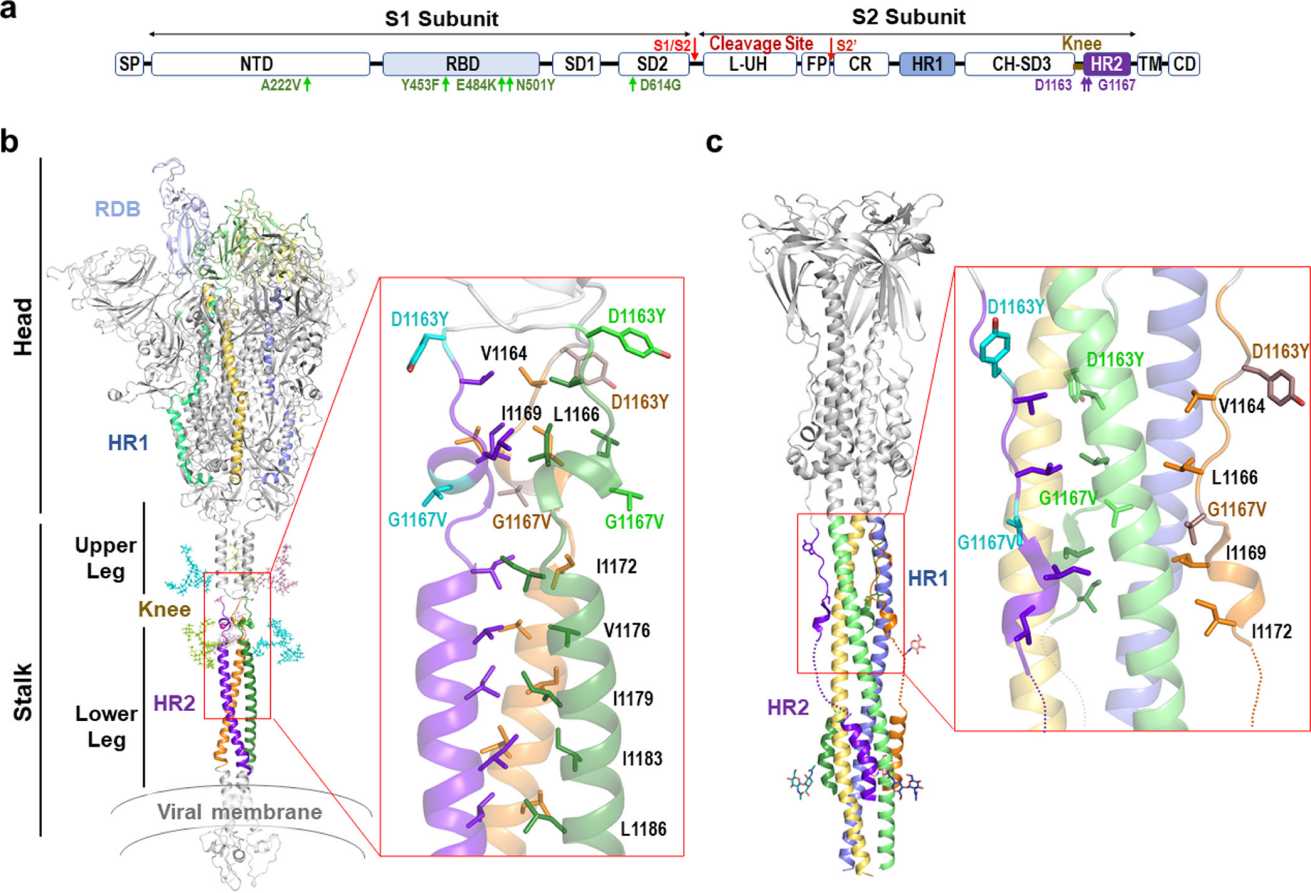
Structure of 1163 and 1167 in the pre- and postfusion states of S protein. (a) Schematic representation of the S protein. SP, signal peptide; NTD, N-terminal domain; RBD, receptor-binding domain; SD1 and SD2, subdomains 1 and 2; L-UH, linker-upstream helix; FP, fusion peptide; CR, connecting region; HR1, heptad repeat 1; CH-SD3, central helix subdomain 3; BH, β-hairpin; HR2, heptad repeat 2; TM, transmembrane; CD, cytoplasmic domain. Amino acid changes D1163Y and G1167V are indicated in purple, and other mutations described in the text are in green. (b) (Left) Cartoon representation of a structural model of prefusion membrane-bound trimeric S protein (77). In each subunit, the RBD, HR1, and HR2 domains are colored in different tones (light to dark) of blue, yellow, and green. The N-glycosylation of N1155 and N1176 is shown in stick representation and colored as the corresponding subunit. Functional and structural regions are marked. (Right) Close-up view of the N-terminal portion of HR2 where D1163Y and G1167V amino acid replacements are found. The side chains of mutated and hydrophobic residues in the HR2 region are shown in stick representation and colored as the corresponding subunit (mutated residues in a lighter tone). (c) Cartoon representation (left) of S2 subunit in postfusion conformation with HR1 and HR2 regions colored as in panel b and N-glycosylation around mutation position shown as sticks. (Right) Close-up view of the region encompassing the mutations (right), showing in stick representation the mutated and hydrophobic residues from the HR2 region shown in panel b. Dotted lines highlight HR2 disordered regions in the cryo-electron microscopy structure.

The S protein mediates both binding to cellular receptors and entry into the host cells (10). For the former, the RBD motif in the S1 subunit interacts with the cellular receptor in the prefusion state. In the postfusion state, two heptad repeat sequences (HR1 and HR2) in the S2 subunit must form a six-helix bundle in order to bring the viral and cellular membrane into close proximity (39, 40) (Fig. 3). S protein positions 1163 and 1167 are both located within the HR2 domain. Specifically, 1167 is present at the beginning of the HR2 motif and 1163 in its upstream linker region (Fig. 3a). Interestingly, this motif is highly invariable, showing 100% conservation across 14 viruses in the subgenus *Sarbecovirus*, to which SARS-CoV-2 belongs (Table S1) (41, 42). Structural characterization of the full-length ectodomain of S protein has shown that the stalk portion encompassing positions 1163 and 1167 presents intrinsic flexibility in the prefusion state (43), precluding its atomic visualization. This was recently confirmed by high-resolution cryo-electron tomographic reconstitution of SARS-CoV-2 (12), where this region was observed to constitute a flexible hinge that acts as a “knee”, connecting two helical coiled-coil regions of the stalk (upper and lower legs) (Fig. 3b). Within this structure, the conformational freedom provided by the glycine residue at position 1167 should play a key role in the flexibility of the knee. In contrast, in the postfusion state, this region shows high rigidity due to a strong structural rearrangement of the HR2 motif, which adopts an extended conformation and tightly packs along the central 3-helix bundle stem formed by the HR1 motif (Fig. 3c). The resulting HR1-HR2 bundle plays a key role in the mechanism of viral-host membrane fusion (43, 44), and mutations in this region could have a significant impact on the function of the S protein. In addition, the HR2 region is highly glycosylated, with this modification being regularly spaced in both the pre- and postfusion states and mostly aligning to the side of the helix bundle (12, 43, 44). Of note, two of these branched sugars are placed at positions N1158 and N1173, shielding positions 1163 and 1167 (Fig. 3b). Therefore, changes in stalk flexibility might have relevance in immunity by influencing both the intrinsic degree of exposure of this region and its sugar shielding.

10.1128/mBio.02315-21.2 TABLE S1 Accession numbers for analyzed sarbecoviruses and SARS-CoV-2 sequences for each data set. Download Table S1, TXT file, 19.2 MB. Copyright © 2021 Ruiz-Rodriguez et al. 2021 Ruiz-Rodriguez et al. https://creativecommons.org/licenses/by/4.0/ This content is distributed under the terms of the Creative Commons Attribution 4.0 International license.

Using the available structural information of the S protein in the pre- and postfusion conformations (43), we examined the possible implications of these mutations to viral infectivity. Based on these structures, G1167V amino acid replacement is predicted to confer significant rigidity to the structure in two ways. First, the introduction of a side chain strongly reduces the conformational freedom provided by the glycine residue. Second, the presence of the new aliphatic side chain provided by the valine residue strongly increases hydrophobicity, likely promoting the burial of this side chain in the HR1 helix 3-bundle stem in the postfusion state or favoring its integration in the neighbor helical coiled-coil in the prefusion state (Fig. 3b and c). Unlike position 1163, position 1167 is fully exposed to the solvent in both the pre- and postfusion states (Fig. 3b and c). Hence, the effect of D1163Y is likely to stem from a change in the nature of the side chain, switching from a charged aspartic acid residue at physiological pH to a polar group with hydrophobic properties in the tyrosine.

### Spike amino acid changes D1163Y and G1167V do not increase viral infectivity.

**FIG 4:**
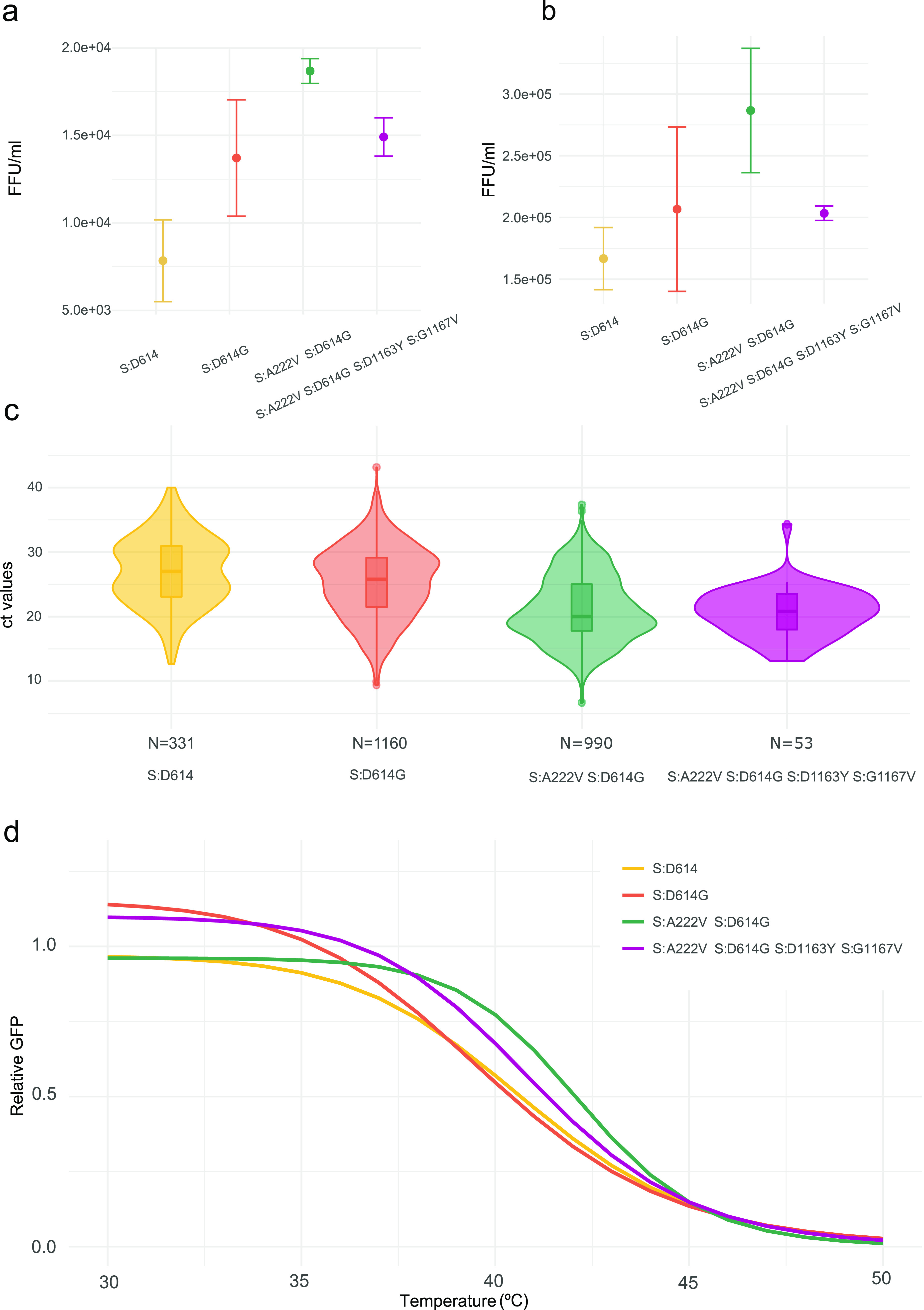
Comparison of the infectivity and stability of different S genotypes. (a and b) The infectivity of VSV particles pseudotyped with each S protein genotype in either Vero cells (a) or human A549 cells expressing ACE2 and TMPRSS2 (b). Means and standard deviations for three replicates are plotted. (c) Comparison of cycle threshold (*C_T_*) values for the N gene from patients infected with viruses encoding different S protein variants. Data are derived from 2,534 sequences from the SeqCOVID consortium. The number of observations (N) analyzed for each genotype is indicated. (d) The thermal sensitivity of VSV pseudotyped with different S genotypes following incubation at 15 min. Data are standardized to the surviving fraction following incubation at 30°C, and the three-parameter log-logistic equation is plotted. FFU, focus forming units.

Previous reports have indicated that mutations in the S protein can increase infectivity (4, 18, 45–47). Because the biggest transmission cluster for amino acid changes in S positions 1163 and 1167 corresponds to the double mutation D1163Y/G1167V (characteristic of 1163.7), we explored whether these amino acid changes in combination influence infectivity. For this, we pseudotyped vesicular stomatitis virus lacking its glycoprotein and encoding green fluorescent protein (48) (VSVΔG-GFP) with different S genotypes: (i) Wuhan S genotype (the reference sequence from Wuhan encoding S:D614), (ii) Wuhan S genotype with S:D614G, (iii) S genotype common in 20E sequences characterized by S:A222V and S:D614G, and (iv) cluster 1163.7 (characterized by S:A222V, S:D614G, S:D1163Y, and S:G1167V). Infectious virus production was then assessed by limiting dilution and counting of GFP-positive cells in both Vero and A549-hACE2-TMPRSS2 cells. As previously reported (16, 18, 49), the 20E S genotype enhanced infectivity relative to the D614 S genotype by 70% in both Vero cells (*P* = 0.005 by unpaired *t* test) (Fig. 4a) and A549-hACE2-TMPRSS2 cells (*P* = 0.016 by unpaired *t* test) (Fig. 4b). The 20E S genotype also showed a trend toward increased infectivity versus the S:D614G replacement alone (35% increase in both cell lines), as previously reported (47), yet the difference was not statistically significant (*P* = 0.2 by unpaired *t* test) (Fig. 4a and b). In contrast, the 1163.7 S genotype significantly diminished virus infectivity versus the 20E genotype, reducing virus titers by 20% in Vero cells (*P* = 0.009 by unpaired *t* test) (Fig. 4a) and 29% in A549-hACE2-TMPRSS2 cells (*P* = 0.03 by unpaired *t* test) (Fig. 4b). This is in agreement with a potential stabilization of the HR2 helix (Fig. 3), which should limit the ability of the S protein to sample different structural conformations that might be required for binding host receptors. Hence, the 1163.7 S genotype does not increase infectivity *in vitro*.

To corroborate that the 1163.7 S genotype does not show higher infectivity *in vivo*, we tested if individuals infected with 1163.7 had higher viral loads. For this, we used the cycle threshold (*C_T_*) of real-time PCR used for diagnosis as a surrogate. As previously reported (16), we detected higher *C_T_* values for the D614 wild-type variant (mean *C_T_* = 27.00) than for genotypes encoding S:D614G (*C_T_* mean = 25.32; *P* < 0.01 by unpaired Wilcoxon test) (Fig. 4c). We did not find significant differences in viral loads between individuals infected with the 1163.7 genotype and other genotypes within 20E (mean *C_T_* = 21.14 versus 20.63; *P* = 0.72 by unpaired Wilcoxon test) (Fig. 4c), in agreement with the lack of infectivity advantage observed *in vitro*. Interestingly, higher viral loads were observed in individuals infected with 1163.7 and other 20E viruses (S:A222V and S:D614G) than the S:D614G virus alone (S:D614G mean *C_T_* = 25.32; 20E mean *C_T_* = 21.14; 1163.7 mean *C_T_* = 20.63; *P* < 0.01 for both comparisons by unpaired Wilcoxon test) (Fig. 4c).

### Amino acid changes D1163Y and G1167V do not alter S protein stability.

As increased spike stability could impact transmissibility by maintaining virion infectivity during the intrahost transmission period, we assessed the temperature sensitivity of the different S variants. For this, we subjected VSV particles pseudotyped with different S genotypes to a range of temperatures for 15 min, after which we evaluated the surviving fraction. Overall, no major differences in the degree to which the different S proteins lost infectivity upon heat exposure were observed, with all S proteins showing a 50% reduction in infectivity at a similar temperature range (39.8 to 42.2°C; *P* > 0.05 for all except Wuhan S genotype [D614] versus 20E S genotype [S:A222V and S:D614G], where *P* is 0.01) (Fig. 4d).

### S:D1163Y and S:G1167V modestly reduce sensitivity to neutralization by existing antibody immunity.

**FIG 5:**
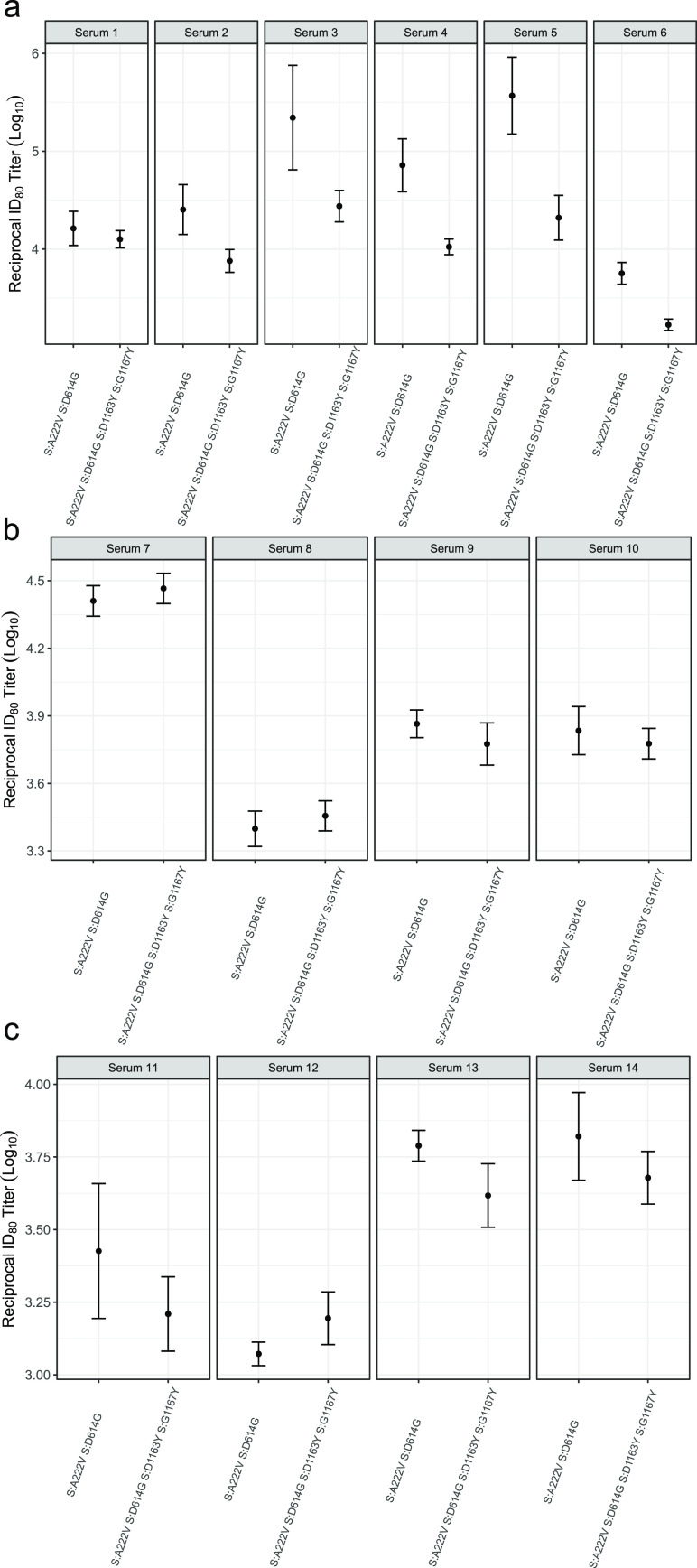
Antibody neutralization of 20E and 1163.7 variants. The reciprocal titer at which infection with the 20E S genotype (S:A222V and S:D614G) or 1163.7 S genotype (20E plus S:D1163Y and S:G1167V) is reduced by 80% (ID_80_) by sera from individuals infected during the early stage of the pandemic (a) or during a later stage of the pandemic (b) and from donors vaccinated with the BNT162b2 vaccine (c). The means and standard errors for three replicates are plotted.

Positions 1163 and 1167 of the S protein have been reported to occur in both T- and B-cell SARS-CoV-2 epitopes (50–52). Moreover, numerous studies have shown that mutations in the S protein can affect antibody neutralization (53, 54). We therefore examined if the presence of D1163Y and G1167V alters the neutralization capacity of convalescent-phase sera using VSV pseudotyped with either the 20E or 1163.7 S genotypes. We tested the sensitivity of these pseudotyped viruses to neutralization by sera from early (April 2020; first wave in Spain) or later (October 2020; second wave in Spain) in the pandemic, when newer variants were dominant (5, 30). Overall, the 1163.7 genotype conferred a modest but statistically significant reduction in sensitivity to neutralization by six serum samples tested from the early stage of the pandemic, as measured by the titers required to inhibit viral entry by 80% (ID_80_; mean = 6.75; range, 1.30 to 17.68; *P* = 0.008 by paired *t* test) (Fig. 5a). A statistically significant but smaller effect was observed when the titers required to inhibit viral entry by 50% were examined (ID_50_; mean = 2.27; range,1.61 to 3.54; *P* < 0.001 by paired *t* test) (Fig. S6). In contrast, both 20E and 1163.7 were equally susceptible to sera from patients infected during the second wave (ID_80_; mean = 1.03; range, 0.87 to 1.23; *P* = 0.83 by paired *t* test) (Fig. 5b). As a modest reduction in titers was observed with sera from early in the pandemic (Fig. 5a), when the S genotype of circulating viruses was more similar to the one present in currently approved vaccines (55, 56), we examined if the 1163.7 S genotype resulted in reduced neutralization by sera from donors vaccinated with the BNT162b2 vaccine. No significant differences in susceptibility to antibody neutralization from vaccinated donors were observed between the two genotypes (Fig. 5c).

10.1128/mBio.02315-21.10 FIG S6 Neutralization of the different mutated S protein variants by convalescent-phase sera from six individuals infected during the first epidemic wave. The reciprocal titer at which each of the different convalescent-phase sera neutralizes the different variants by 50% is indicated. Data are means and standard errors (*n* = 3). Download FIG S6, PDF file, 0.08 MB. Copyright © 2021 Ruiz-Rodriguez et al. 2021 Ruiz-Rodriguez et al. https://creativecommons.org/licenses/by/4.0/ This content is distributed under the terms of the Creative Commons Attribution 4.0 International license.

## DISCUSSION

SARS-CoV-2 success is linked to its ability to infect and be transmitted. Mutations that emerge independently several times and increase in frequency are likely to confer enhanced viral infectivity, transmission, or immune evasion. The identification of such mutants is of great importance, as they can significantly impact public health. On the other hand, the appearance of mutations can also be driven by stochastic events, and the ability to evaluate the potential risk posed by new variants is of key importance to appropriately tailor public health responses. In this work, we identified two amino acid replacements at positions 1163 and 1167 of the S protein that appeared to be potentially beneficial for the virus based on several lines of evidence. First, these mutations are highly variable within SARS-CoV-2 but conserved across the closely related coronaviruses. Second, the vast majority of sequences harboring these mutations appeared in clusters (Fig. 1a and b). Third, both positions have been reported as positively selected multiple times throughout the SARS-CoV-2 phylogeny indicating a fitness advantage (57). Finally, the largest cluster containing either of these mutations, and therefore the most successful in terms of transmission, harbored both mutations together (Fig. 1a and b). This infection cluster was sustained for more than 6 months across Europe, suggesting that both mutations together could increase viral fitness.

For these reasons, we conducted a series of experiments to assess whether the two mutations conferred a biological advantage to the virus *in vitro*. Analysis of the mutation in the context of available structures suggested that G1167V could alter the flexibility of the S protein stalk by both restricting the conformational freedom normally conferred by the wild-type glycine residue and by introducing a hydrophobic side chain that will favor burial in the HR2 coiled-coil leucine zipper of the prefusion state (Fig. 3). This extensive flexibility of the S prefusion stalk seems to be unique to the SARS-CoV-2 (43) and has been suggested to increase avidity for the host receptors by allowing the engagement of multiple S proteins (43). Therefore, stalk stabilization by G1167V is likely to result in a reduced ability of S to bind receptors in the target cell. In agreement with this, we found reduced infectivity upon introduction of both changes D1163Y and G1167V into the spike protein (Fig. 4a and b). In addition, we found no indication of resistance to heat inactivation that could facilitate environmental transition between hosts (Fig. 4c), and the viral load in clinical specimens showed no difference due to the presence of these two mutations compared to the 20E S genotype (Fig. 4d).

We examined if these two mutations conferred evasion of preexisting immunity, which could compromise vaccine efficacy and/or result in reinfection. For this, we used sera from both the first (April 2020) and second (October 2020) epidemic waves of the infection in Spain, because an almost complete replacement of SARS-CoV-2 S genotypes of different variants occurred between these two time points in Spain (30). When utilizing sera from donors infected during the first wave of the pandemic in Spain, we found a modest but statistically significant reduction in susceptibility to neutralization of the 1163.7 S genotype compared to the 20E S genotype of approximately 6-fold (Fig. 5a). However, no difference in neutralization was observed between the two variants when sera from patients infected during the second wave were used (Fig. 5b). Overall, the magnitude of the observed reduction in neutralization susceptibility to sera from individuals infected during the first wave was much less pronounced than that observed for other genotypes implicated in immune evasion (54), although the degree of reduced neutralization required to confer a biologically relevant fitness advantage *in vivo* has not been established. Importantly, we also found no evidence for reduced neutralization of the 1163.7 variant by sera from donors immunized with the BNT162b2 vaccine (Fig. 5c). Since all currently available vaccines, including BNT162b2, are based on the Wuhan S genotype, it is expected that these mutations will not reduce the effectiveness of the other vaccines either.

Both S amino acid positions 1163 and 1167 are embedded in experimentally confirmed T- and B-cell epitopes. Interestingly, for T-cell epitopes, a predicted HLA-II epitope including positions 1163 and 1167 has been experimentally verified to bind to HLA DRB1*01:01, the prototype molecule for the DR supertype (epitope identifier in Immune Epitope DataBase: 9006 [58]). Additionally, amino acid S:D1163 is included in a SARS-CoV-2 T-cell linear epitope eliciting T-cell responses in convalescent COVID-19 cases (59) as well as in SARS-CoV-2-naive individuals (52), indicating cross-reactivity in epitopes involving these regions. B-cell linear epitopes that span D1163 and G1167 have also been reported (51), with D1163 belonging to a dominant linear B-cell epitope recognized by more than 40% COVID-19 patients used in the assay (53). Hence, it is possible that these mutations could play a role in modulating T-cell responses. However, at the time cluster 1163.7 appeared and transmitted in Europe, large-scale vaccination had not been implemented and the majority of the population had not been infected by SARS-CoV-2. Therefore, there was likely little selection of SARS-CoV-2 variants that evade existing immunity.

Overall, clinical and experimental data do not support the idea that D1163Y and G1167V in the S protein confer temperature resistance, higher infectivity *in vitro*, higher viral load *in vivo*, or significant escape from antibody neutralization. The biological consequences of these mutations are therefore unlikely to confer a significant fitness advantage. Indeed, these early findings are in agreement with the subsequent observation that these mutations ceased to circulate in Europe as VOC Alpha increased in frequency.

## MATERIALS AND METHODS

### Whole-genome sequencing and genome assembly of SeqCOVID consortium sequences.

A total of 5,017 clinical samples were received, sequenced, and analyzed by the SeqCOVID consortium from all autonomous communities of Spain. These samples were confirmed as SARS-CoV-2 positive by reverse transcription-PCR (RT-PCR) carried out by clinical microbiology services from each hospital. All sequences are available at GISAID under the accession numbers detailed in Table S1.

For sequencing, RNA samples were retrotranscribed into cDNA. SARS-CoV-2 complete genome amplification was performed in two multiplex PCRs, according to the protocol developed by the ARTIC network (60), using the V3 multiplex primer scheme (61). From this step, two amplicon pools were prepared, combined, and used for library preparation. The genomic libraries were constructed with the Nextera DNA Flex sample preparation kit (Illumina Inc., San Diego, CA) according to the manufacturer’s protocol, with 5 cycles for indexing PCR. Whole-genome sequencing was performed in the MiSeq platform (2 × 200 cycles paired-end run; Illumina).

Reads obtained were processed through a bioinformatic pipeline based on iVar (58), available at https://gitlab.com/fisabio-ngs/sars-cov2-mapping. The first step in the pipeline removed human reads with Kraken (59); then, fastq files were filtered using fastp (62) v 0.20.1 (arguments employed: –cut tail, –cut-window-size, –cut-mean-quality, -max_len1, -max_len2). Finally, mapping and variant calling were performed with iVar v 1.2, and quality control assessment was carried out with MultiQC (63).

### Analysis of the S gene of sarbecoviruses related to SARS-CoV-2.

Fourteen sequences including SARS-CoV-2 belonging to sarbecoviruses, sequences were annotated with annotation files available in the NCBI database in order to locate the spike gene coordinates (accession numbers are available in Table S1). The 14 sequences harboring the S gene were concatenated and aligned with MEGA-X (64) using amino acids with the ClustalW algorithm with default options.

### Sampling SARS-CoV-2 from non-Spanish consortium sequences.

To build the global alignment, sequences were downloaded from GISAID including all the pandemic periods since the first known case sequenced (from 24 December 2019) until the last sample on 22 December 2020. We used two filters to select the data set: sequences with more than 29,000 bp, and sequences with known dates of sampling. Sequences downloaded from GISAID were aligned against the SARS-CoV-2 reference genome (65) using MAFFT (66), omitting all insertions and getting an alignment length of 29,903 bp. The final alignment constructed included 270,869 sequences, all sequences with GISAID ID used for this study are available in Table S1.

### Frequency and detection of mutated positions.

Single nucleotide variants were detected using the global data set alignment, generating a VCF file with SNP sites (67) v 2.5.1 (argument employed: -v), using the reference genome as the reference bases for detecting mutations. This VCF file was processed with a Python script to assess all mutated samples by position, calculating the frequencies of the global data set and annotating sequences with the detected mutations. After that, the mutated positions were annotated with snpEff (68) v 5.0 using SARS-CoV-2 reference database annotation (arguments employed: -c, -noStats, -no-downstream, -no-upstream, NC_045512.2).

Genotypes detected that involved mutations in 1163 and 1167 such as clusters 1163.7 and 163.654 were represented in a circos plot with the R package circlize (69) v 0.4.12.1004.

### Alignments.

For the phylogenetic analysis, a reduced data set was selected from the 270,869 sequences. Duplicated sequences were removed with seqkit v 0.13.2 (arguments employed: rmdup -s). A total of 8,397 sequences were selected at random with the same temporal distribution by month as the initial data set by Python scripting. The 8,397 sequences were concatenated with 2,053 sequences harboring amino acid replacements in D1163 and G1167 of the S protein, thus resulting in an alignment of 10,450 sequences (Table S1).

The data set to represent Alpha phylogenetic relationships included 3,067 randomly selected samples identified by the PANGO typing system (https://github.com/cov-lineages/pangolin) as 20I plus the 33 sequences with amino acid replacements in S:D1163 and/or S:G1167 (Table S1).

For all the alignments, problematic positions reported by Lanfear (70) were masked for the phylogenetic reconstruction using masked_alignment.sh script.

### Phylogenetic analysis.

Maximum-likelihood phylogenies in Fig. 1 and Fig. S2, S4, and S5 were reconstructed from the masked alignment using IQ-TREE (71) v 1.6.12 with GTR model and collapsing near-zero branches (arguments employed: -czb, -m GTR). The phylogenies were annotated and visualized with iTOL v 4 (72).

10.1128/mBio.02315-21.8 FIG S4 Maximum-likelihood phylogeny of 3,067 genomes belonging to Alpha, rooted with the reference sequence. Mutated sequences with S:D1163 and/or S:G1167 amino acids are colored in the circle. The scale bar indicates the number of nucleotide substitutions per site. The biggest clades are collapsed and represented with isosceles triangles. Download FIG S4, PDF file, 0.08 MB. Copyright © 2021 Ruiz-Rodriguez et al. 2021 Ruiz-Rodriguez et al. https://creativecommons.org/licenses/by/4.0/ This content is distributed under the terms of the Creative Commons Attribution 4.0 International license.

The phylogeny in Video S1, composed by 10,450 sequences, was built with the Nextstrain pipeline (https://github.com/nextstrain/augur) to monitor and visualize temporal and geographical transmission of 1163.7.

### Clusters of transmission involving 1163 and 1167 S amino acid replacements.

We used the phylogeny of 10,450 sequences enriched with all sequences mutated in 1163 and 1167 to quantify the minimum number of mutational events involving positions 1163 and 1167 in the S protein. We first defined which mutations characterize internal nodes using R packages: tidytree v 0.3.3 and treeio v 1.14.3 (73). We then depicted monophyletic clusters sharing at least one of the two mutations. Transmission clusters were defined as all sequences that (i) are derived from an internal node characterized by the same nucleotide mutation involving 1163 or 1167 amino acid replacements, (ii) include more than one sequence, and (iii) have the nucleotide mutation in at least 95% of sequences. Additionally, redundant nodes were eliminated, keeping the ancestral node of the cluster. Sequences with at least one mutation but not in clusters were counted as single events of mutation in the phylogeny.

### Structural analysis of 1163 and 1167 S amino acid replacements.

The atomic coordinates for S protein in prefusion state were retrieved from the CHARMM-GUI COVID-19 Archive (http://www.charmm-gui.org/docs/archive/covid19). The atomic coordinates for S protein in the postfusion state were retrieved from Protein Data Bank (PDB code 6XRA [43] and PDB code 6LXT [74]). Mutations were introduced using single mutation tool embedded in COOT (75), and figures were generated with PyMOL (www.pymol.org).

### Production of SARS-CoV-2-pseudotyped vesicular stomatitis virus, titration, and thermal stability evaluation.

Mutations were introduced into a plasmid encoding a codon-optimized S protein (14) by site directed mutagenesis (see Table S3 for primers). All mutations were verified by Sanger sequencing (see Table S3 for primers). To evaluate the efficiency of virus production, three transfections in HEK293 cells (CRL-1573 from ATCC) were performed for each plasmid to generate pseudotyped VSV harboring the indicated S protein (76). The titers of the virus produced were then assayed by serial dilution, followed by infection of either Vero cells (CCL-81 from ATCC) or A549 cells expressing ACE2 and TMPRSS2 (InvivoGen catalog code a549-hace2tpsa) and counting of GFP-positive cells (focus-forming units [FFU]) at 16 h postinfection. Statistical comparisons were performed by unpaired *t* test (R package: stats v 3.6.1) with normalized logarithmic data. For assessing thermal stability, 1,000 FFU (as measured on Vero cells) were incubated for 15 min at 30.4, 31.4, 33, 35.2, 38.2, 44.8, 47, 48.6, or 49.6°C before addition to Vero cells previously seeded in a 96-well plate (10,000 cells/well). GFP signal in each well was determined 16 h postinfection using an Incucyte S3 system (Essen Biosciences). The mean GFP signal observed in several mock-infected wells was subtracted from those of all infected wells, followed by standardization of the GFP signal to the mean GFP signal from wells incubated at 30.4°C. Finally, a three-parameter log-logistic function was fitted to the data using the drc package v 3.0-1 in R (LL.3 function), and the temperature resulting in 50% inhibition was calculated using the drc ED function. Statistical differences in the temperature resulting in 50% reduction of infection were evaluated using the drc EDcomp function.

10.1128/mBio.02315-21.4 TABLE S3 Primers for site directed mutagenesis of plasmid encoding codon-optimized S protein and for sanger sequencing to detect mutations of interest. Download Table S3, XLSX file, 0.01 MB. Copyright © 2021 Ruiz-Rodriguez et al. 2021 Ruiz-Rodriguez et al. https://creativecommons.org/licenses/by/4.0/ This content is distributed under the terms of the Creative Commons Attribution 4.0 International license.

### Evaluation of neutralization by convalescent-phase sera and efficacy of virus particle production.

Pseudotyped VSV virions bearing the 20E or 1163.7 S genotype were evaluated for sensitivity to neutralization by convalescent-phase sera as previously described (76). Briefly, 16 h postinfection, GFP signal in each well was determined using an Incucyte S3 system (Essen Biosciences). The mean GFP signal observed in several mock-infected wells was subtracted from that of all infected wells, followed by standardization of the GFP signal in each well infected with antibody-treated virus to that of the mean GFP signal from wells infected with mock-treated virus. Any negative values resulting from background subtraction were arbitrarily assigned a low, nonzero value (10^−5^). The serum dilutions were then converted to their reciprocal, their logarithm (log_10_) was taken, and the dose resulting in 50% (ID_50_) or 80% (ID_80_) reduction in GFP signal was calculated in R using the drc package v 3.0-1. A two-parameter log-logistic regression (LL2 function) was used for all samples except when a three-parameter logistic regression provided a significant improvement to fit, as judged by the ANOVA function in the drc package (e.g., *P* < 0.05 following multiple-testing correction using the Bonferroni method). All first-wave samples were obtained from donors that were admitted to the intensive care unit and were collected during April 2020. For the second-wave donors, sera were obtained (October 2020) from patients with severe COVID-19 requiring inpatient treatment. Similarly, samples were obtained from immunized donors who had no history of SARS-CoV-2 infection and who had received a second dose of Pfizer-BioNTech COVID-19 vaccine (BNT162b2; February 2021). All vaccinated individuals tested negative for antibodies against the SARS-CoV-2 N protein using a dual-recognition immunochromatographic assay (INgezim COVID 19 CROM 50.CoV.K41; Eurofins Ingenasa).

### Ethics approval and consent to participate.

Sequencing of the samples was approved by the ethics committee Comité Ético de Investigación de Salud Pública y Centro Superior de Investigación en Salud Pública (CEI DGSP-CSISP), no. 20200414/05.

All samples from Hospital Universitario y Politécnico La Fe de Valencia were collected after informed written consent had been obtained, and the project was approved by the ethical committee and institutional review board (registration number 2020-123-1).

### Data availability.

All generated SARS-CoV-2 genomes from SeqCOVID consortium are available in the GISAID platform under the accession numbers available in Table S1. Code and data used are available at the GitHub repository (https://github.com/PathoGenOmics/1163.7_SARS-CoV-2).

10.1128/mBio.02315-21.1 VIDEO S1 Geographical transmission of 1163.7 visualized with Nextstrain build. Download Movie S1, MOV file, 1.7 MB. Copyright © 2021 Ruiz-Rodriguez et al. 2021 Ruiz-Rodriguez et al. https://creativecommons.org/licenses/by/4.0/ This content is distributed under the terms of the Creative Commons Attribution 4.0 International license.
