# Supplementary figures and images for: Evolutionary and Phenotypic Characterization of Two Spike Mutations in European Lineage 20E of SARS-CoV-2
Source: mBio. 2021 Nov 16;12(6):e02315-21. doi: 10.1128/mBio.02315-21 (PMC8593680; doi:10.1128/mBio.02315-21)

**a**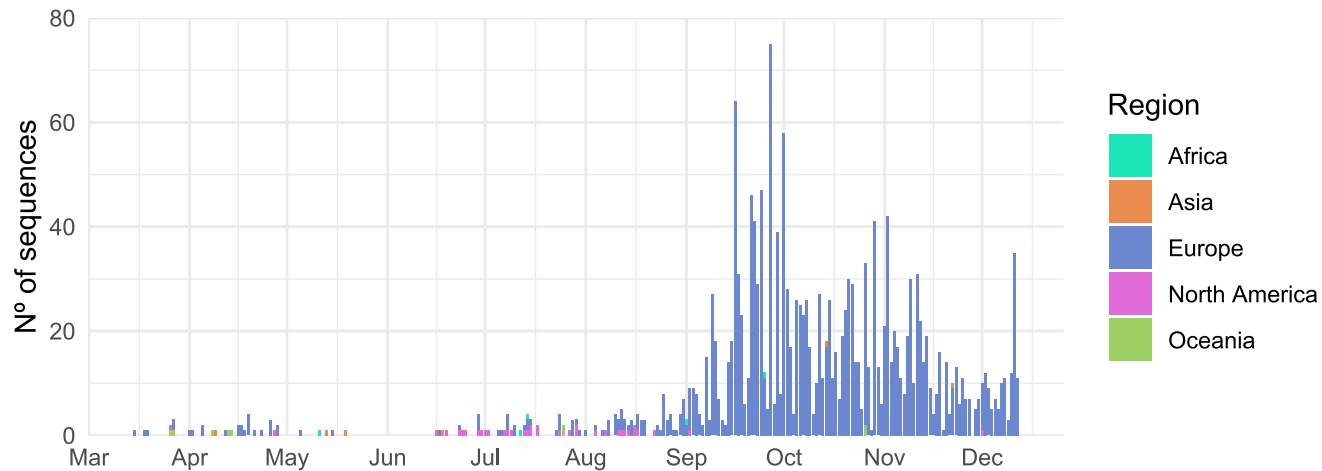**b**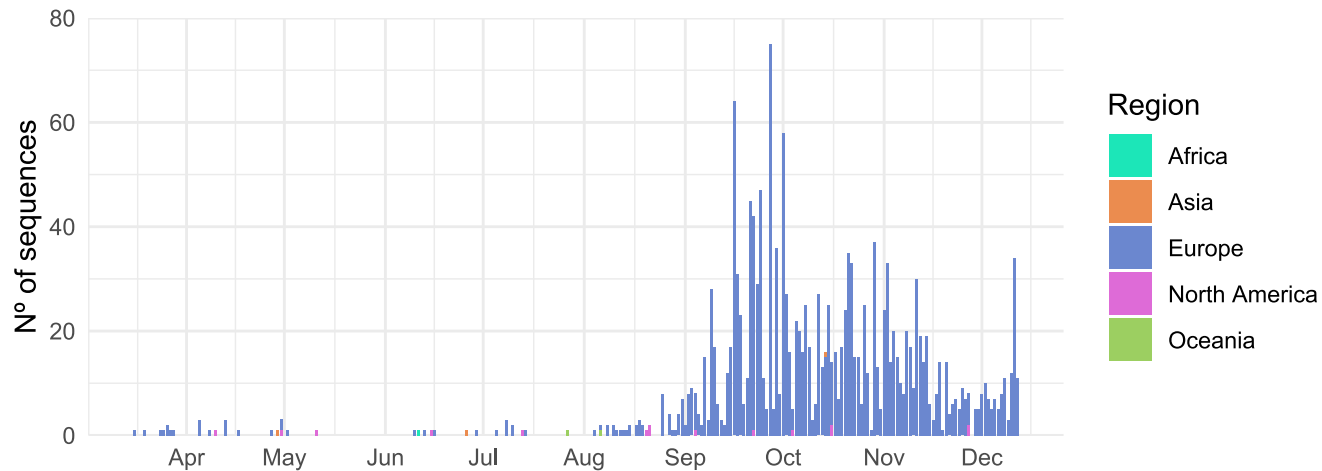

Supplement: FIG S1 [file mbio.02315-21-sf001.pdf]

c

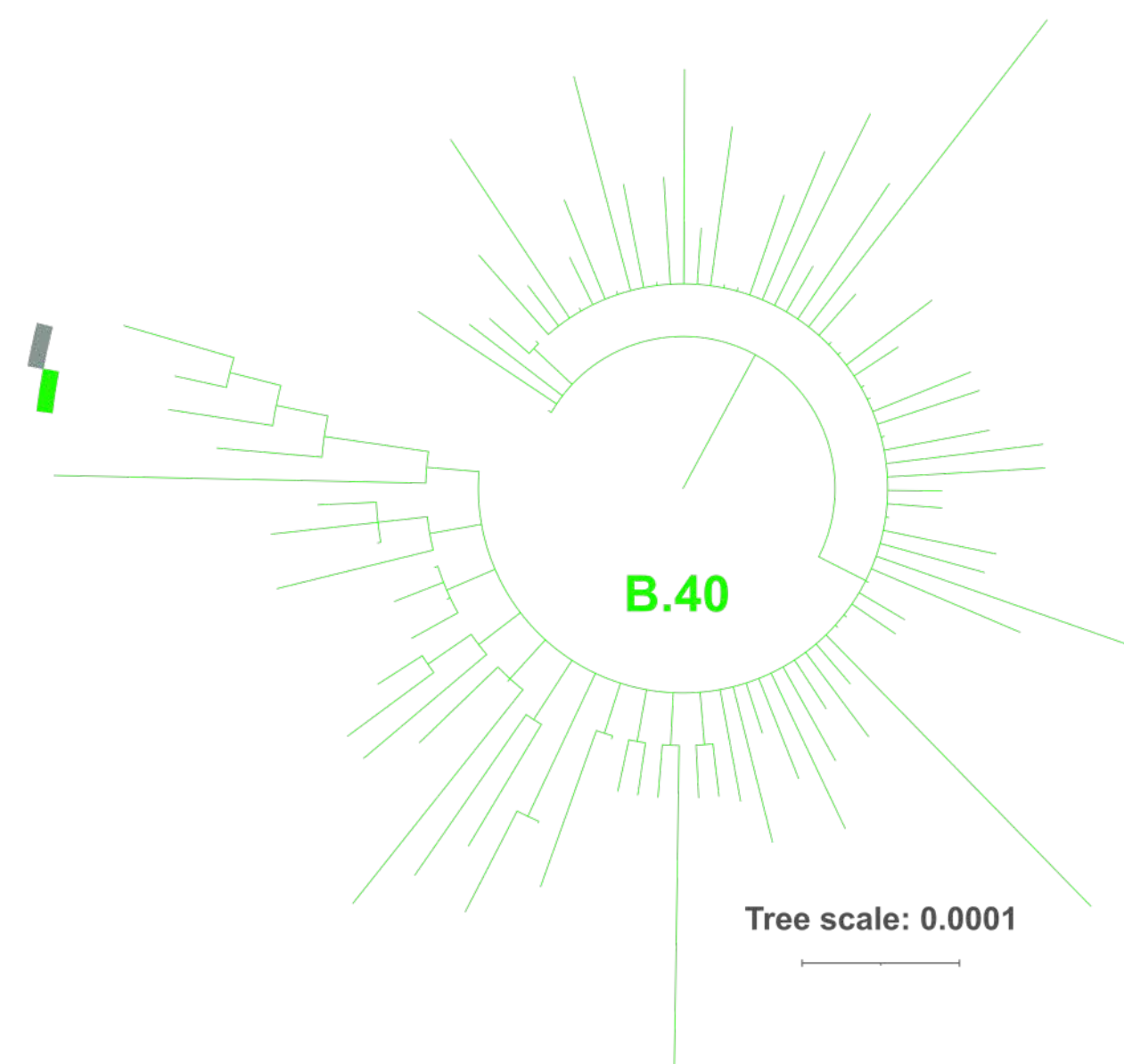

d

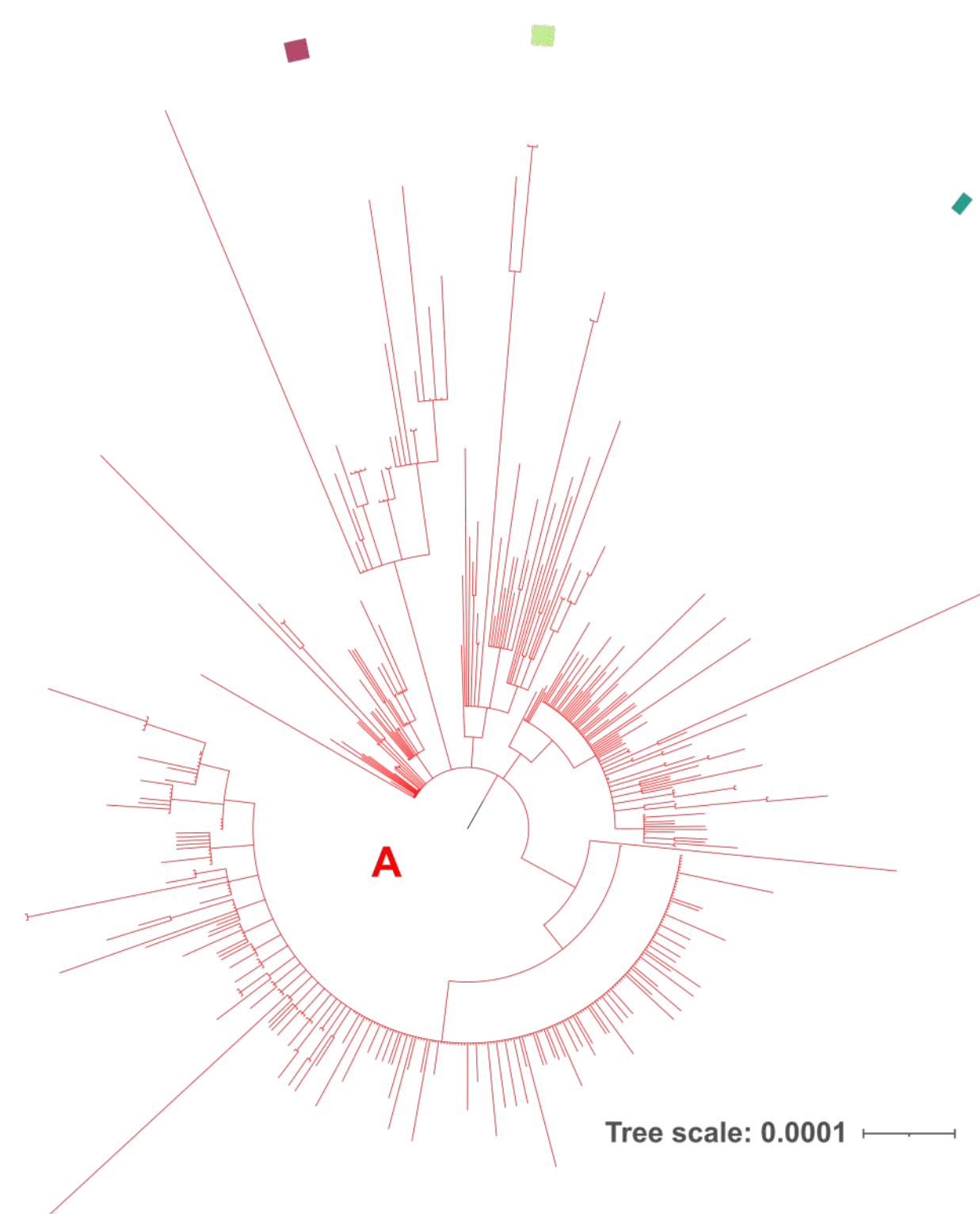

b

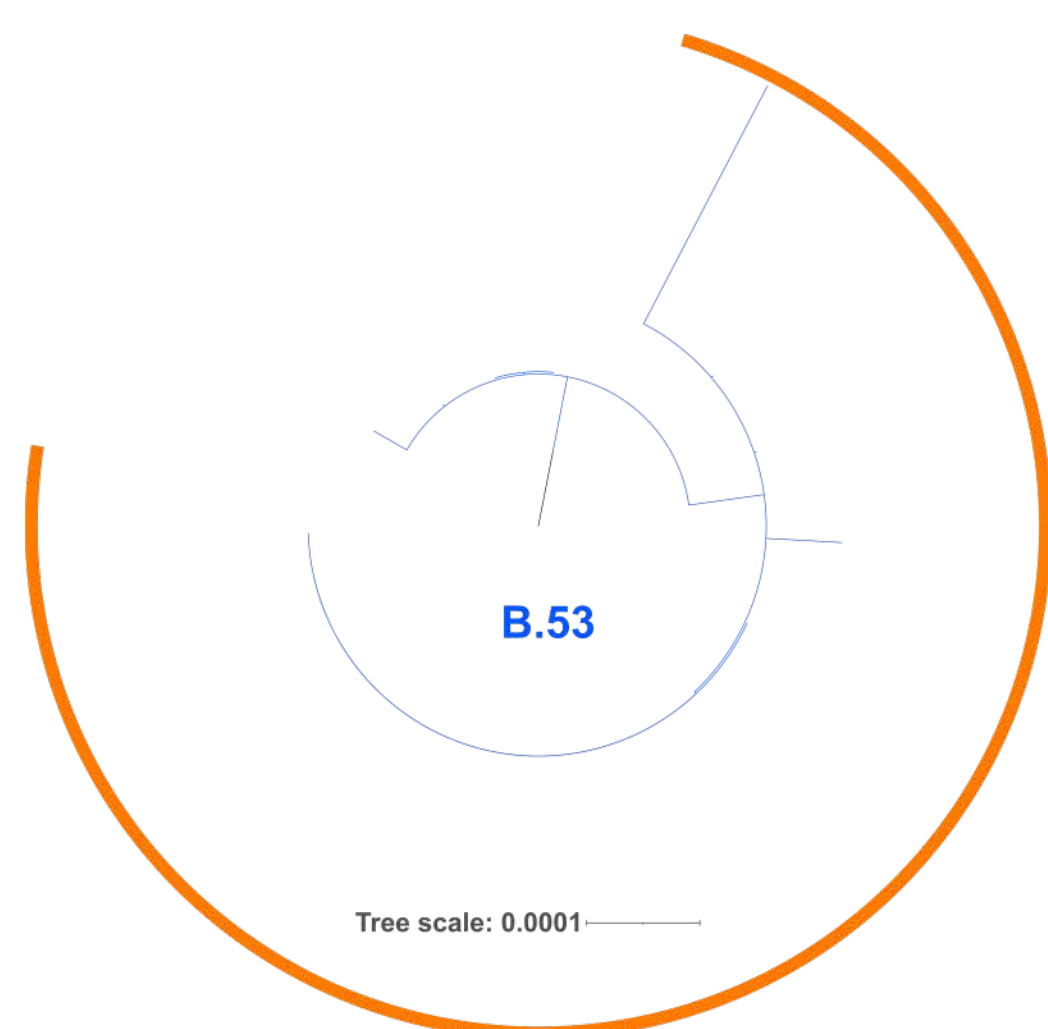

a

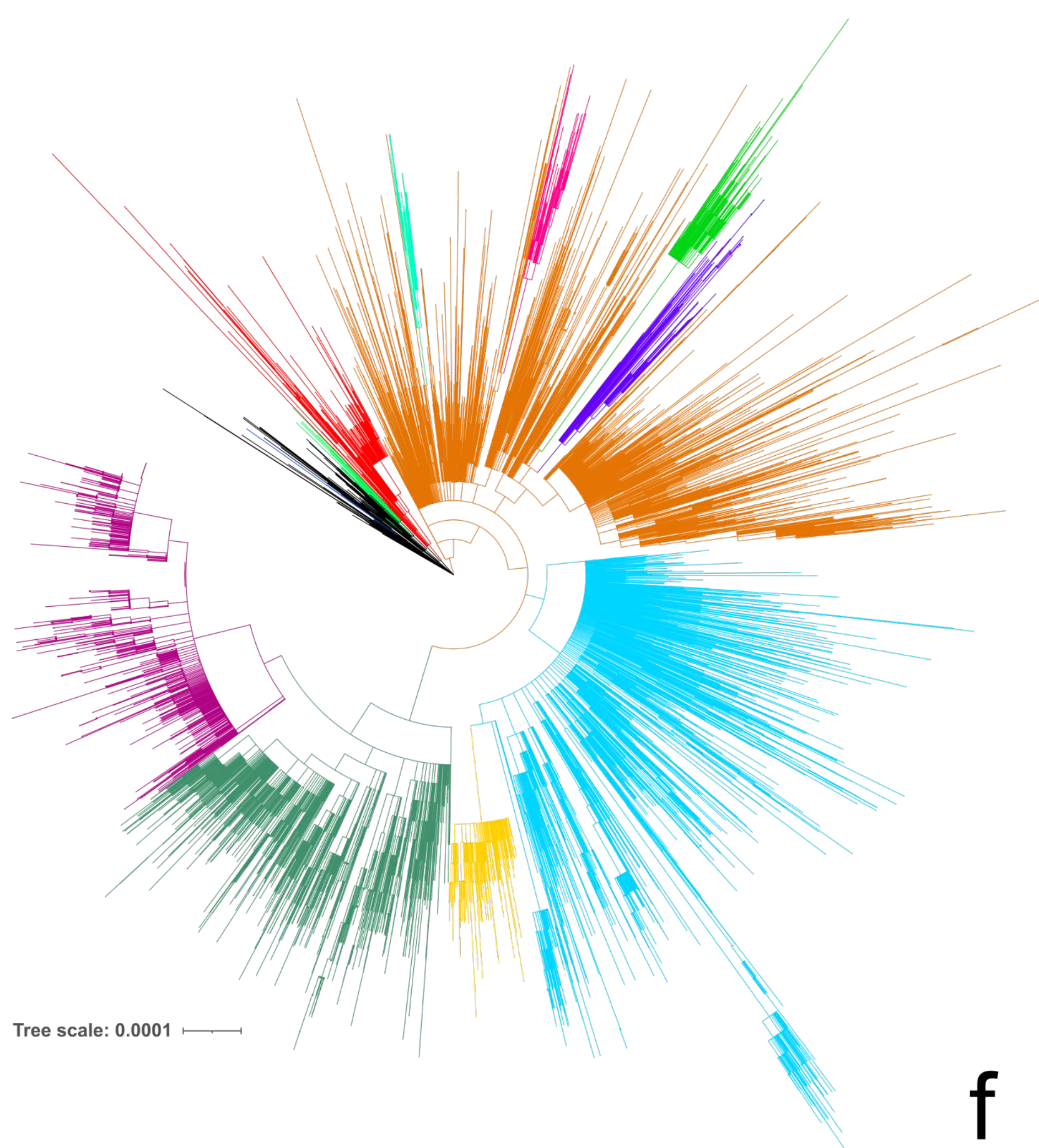

e

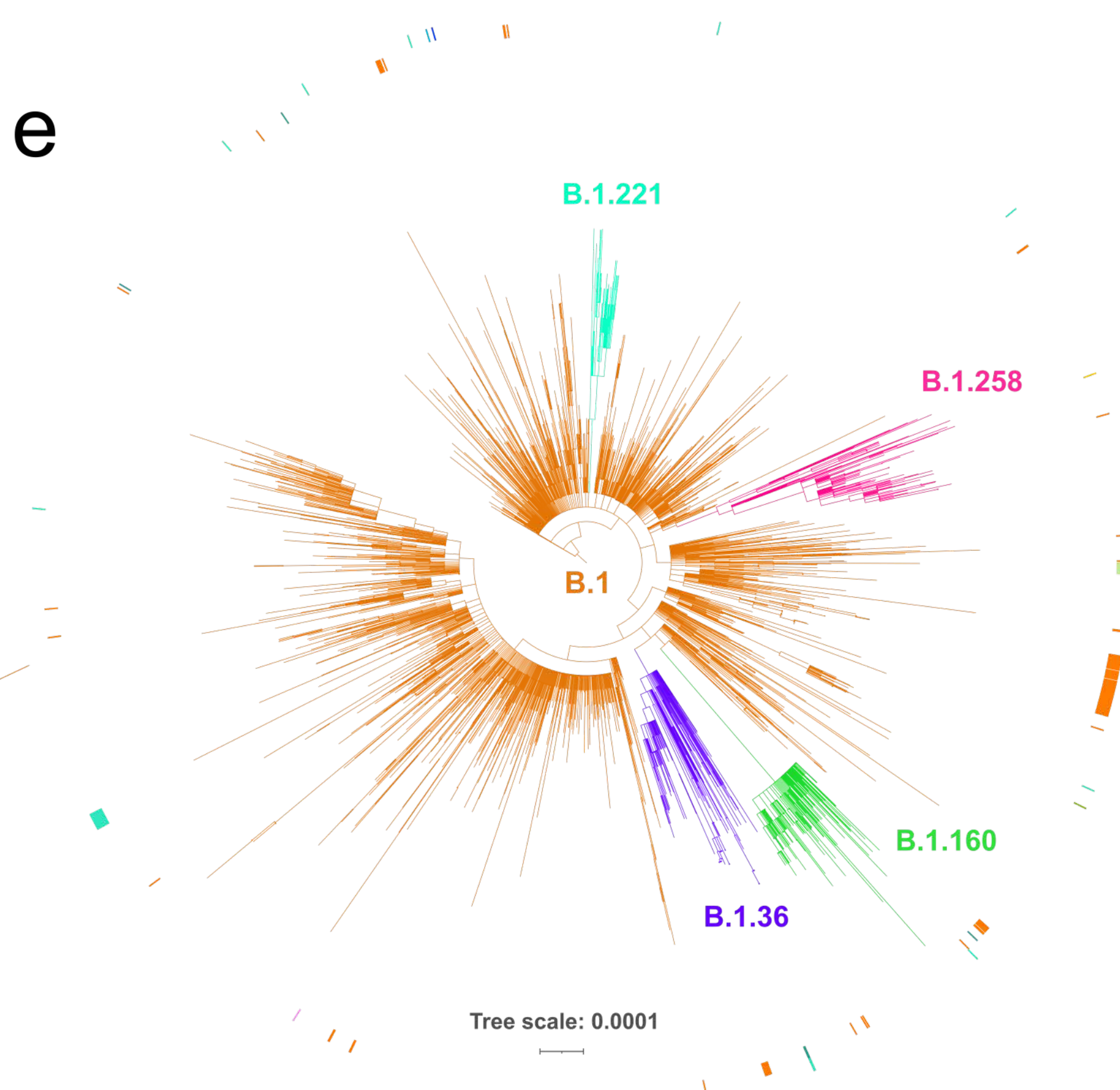

f

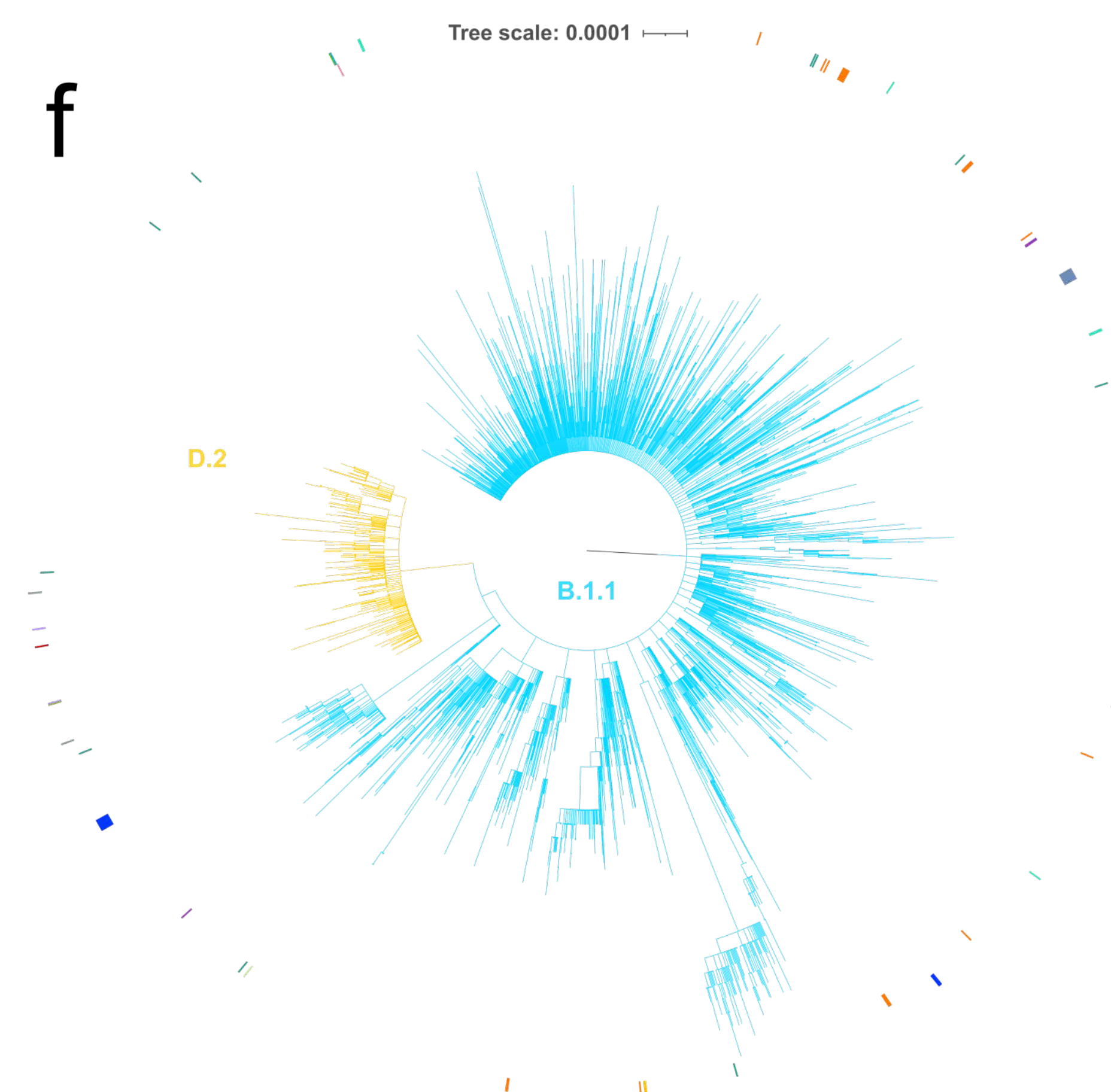

g

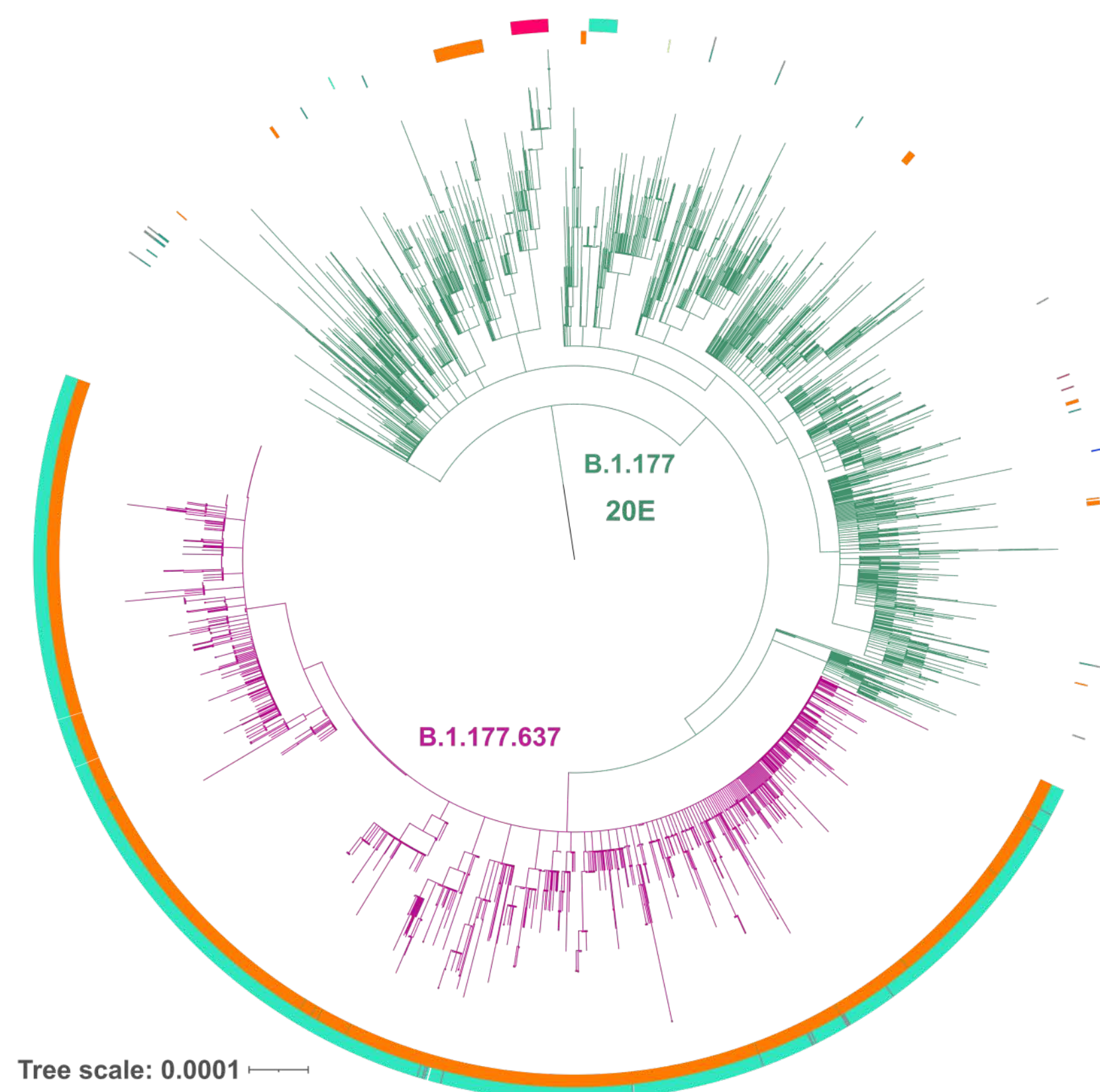

| Position D1163 | Position G1167 |
|----------------|----------------|
| 1163A          | 1167A          |
| 1163E/D        | 1167C/G        |
| 1163G          | 1167D          |
| 1163H          | 1167F          |
| 1163Y/D        | 1167R          |
| 1163H/Y/D      | 1167R/G        |
| 1163N/D        | 1167S          |
| 1163V          | 1167S/G        |
| 1163Y          | 1167V          |
|                | 1167V/G        |

Supplement: FIG S2 [file mbio.02315-21-sf002.pdf]

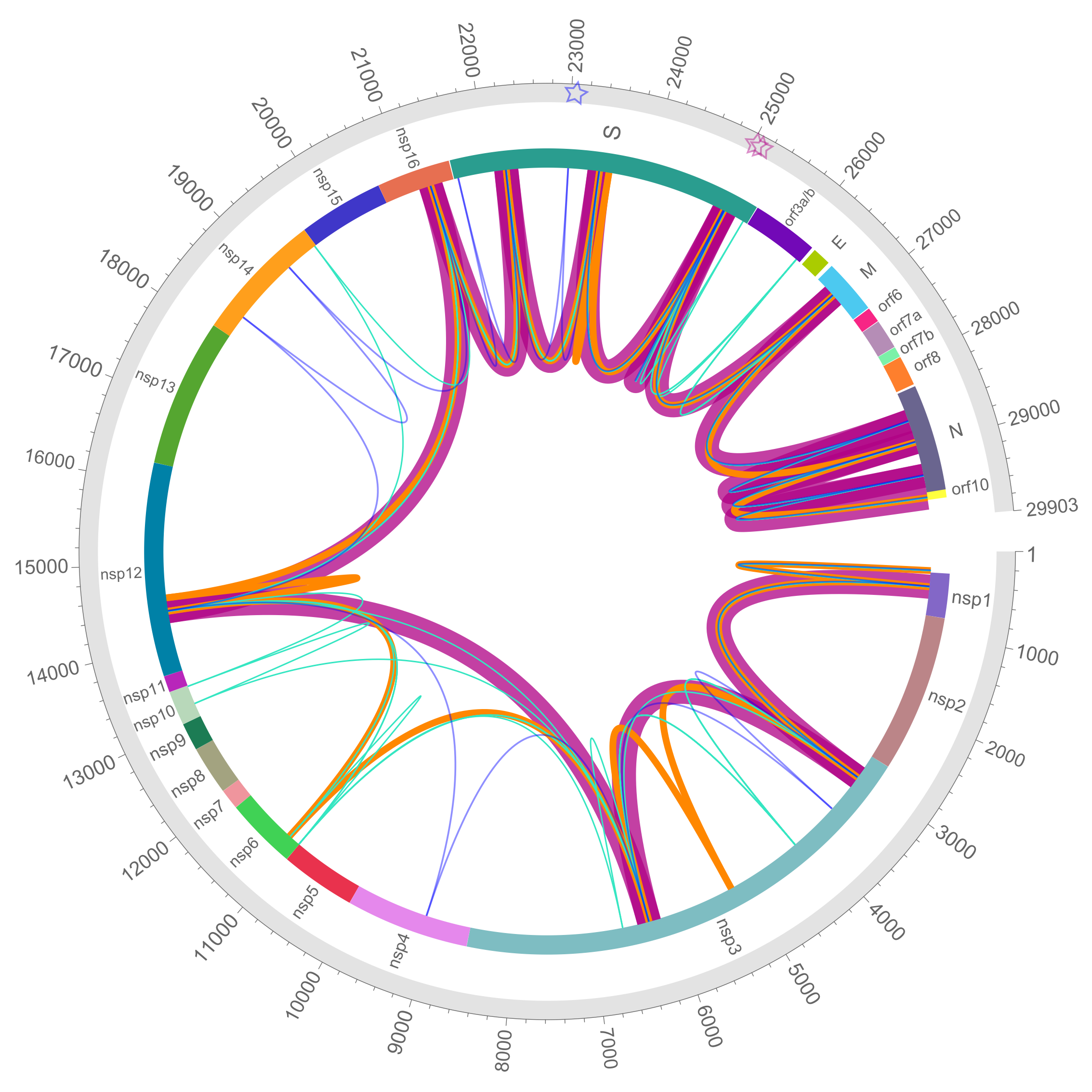

Supplement: FIG S3 [file mbio.02315-21-sf003.pdf]

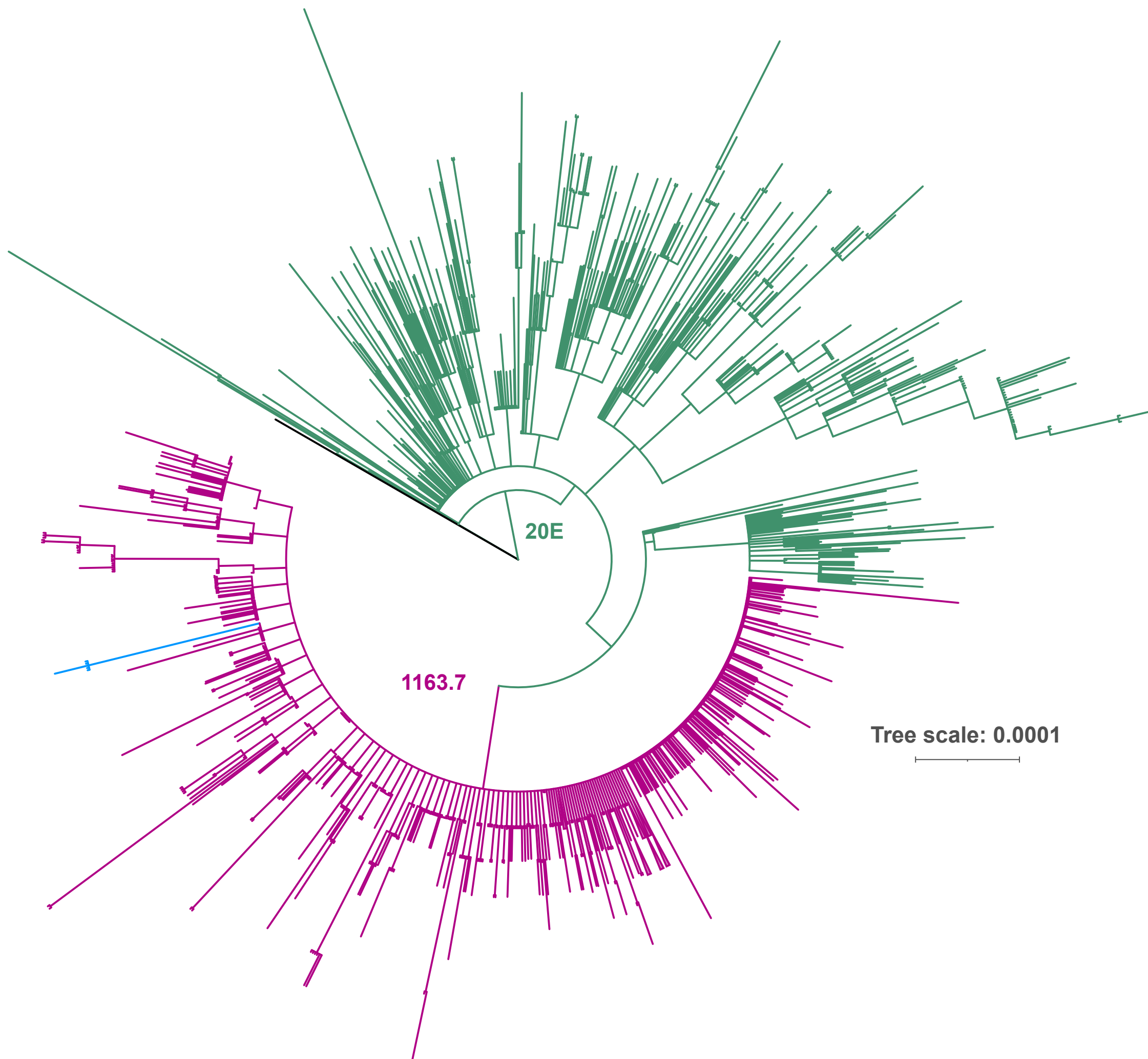

Supplement: FIG S5 [file mbio.02315-21-sf005.pdf]

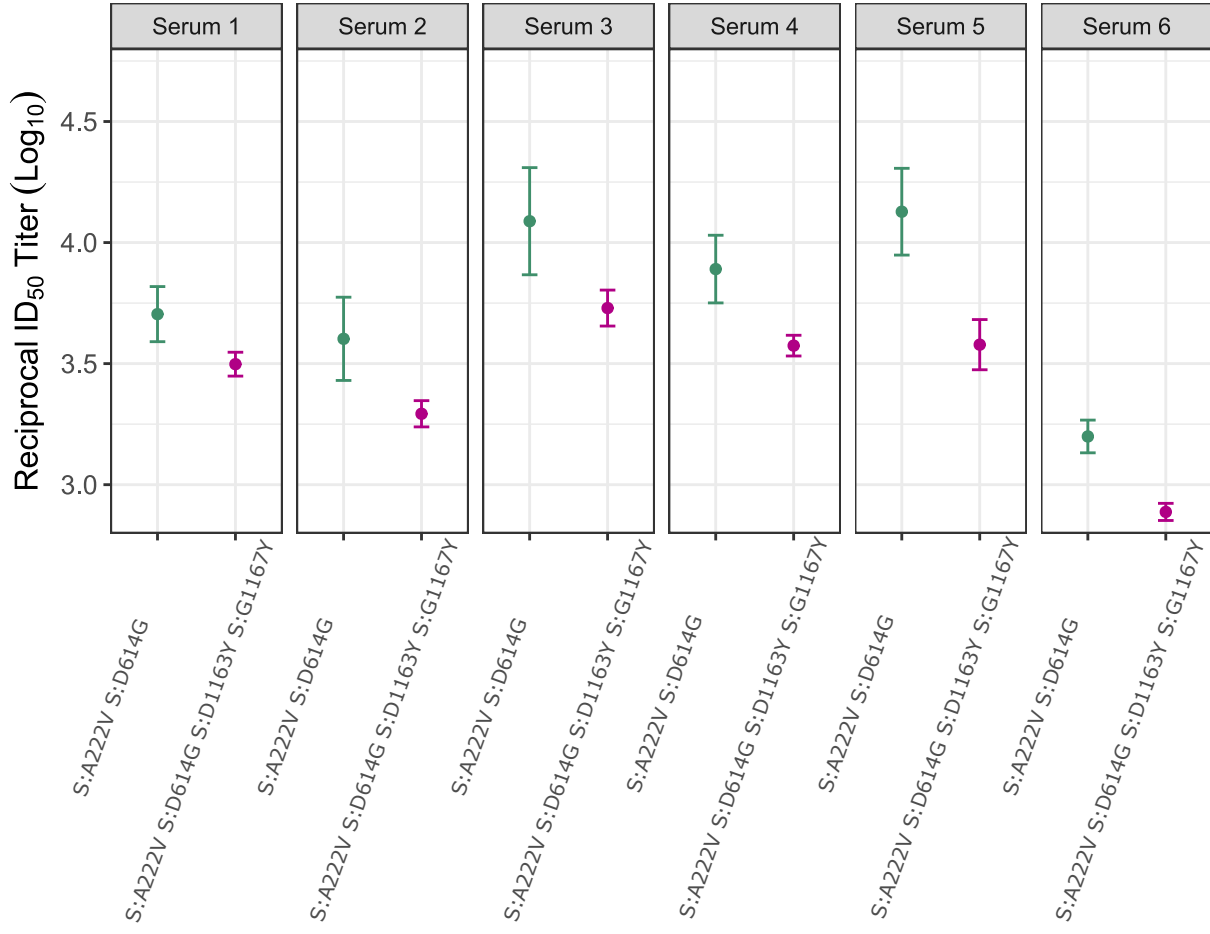

Supplement: FIG S6 [file mbio.02315-21-sf006.pdf]

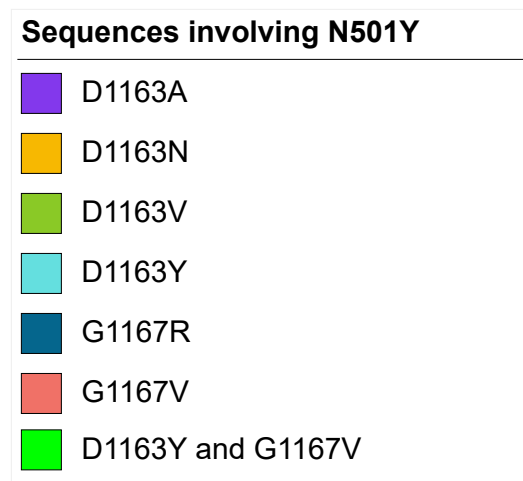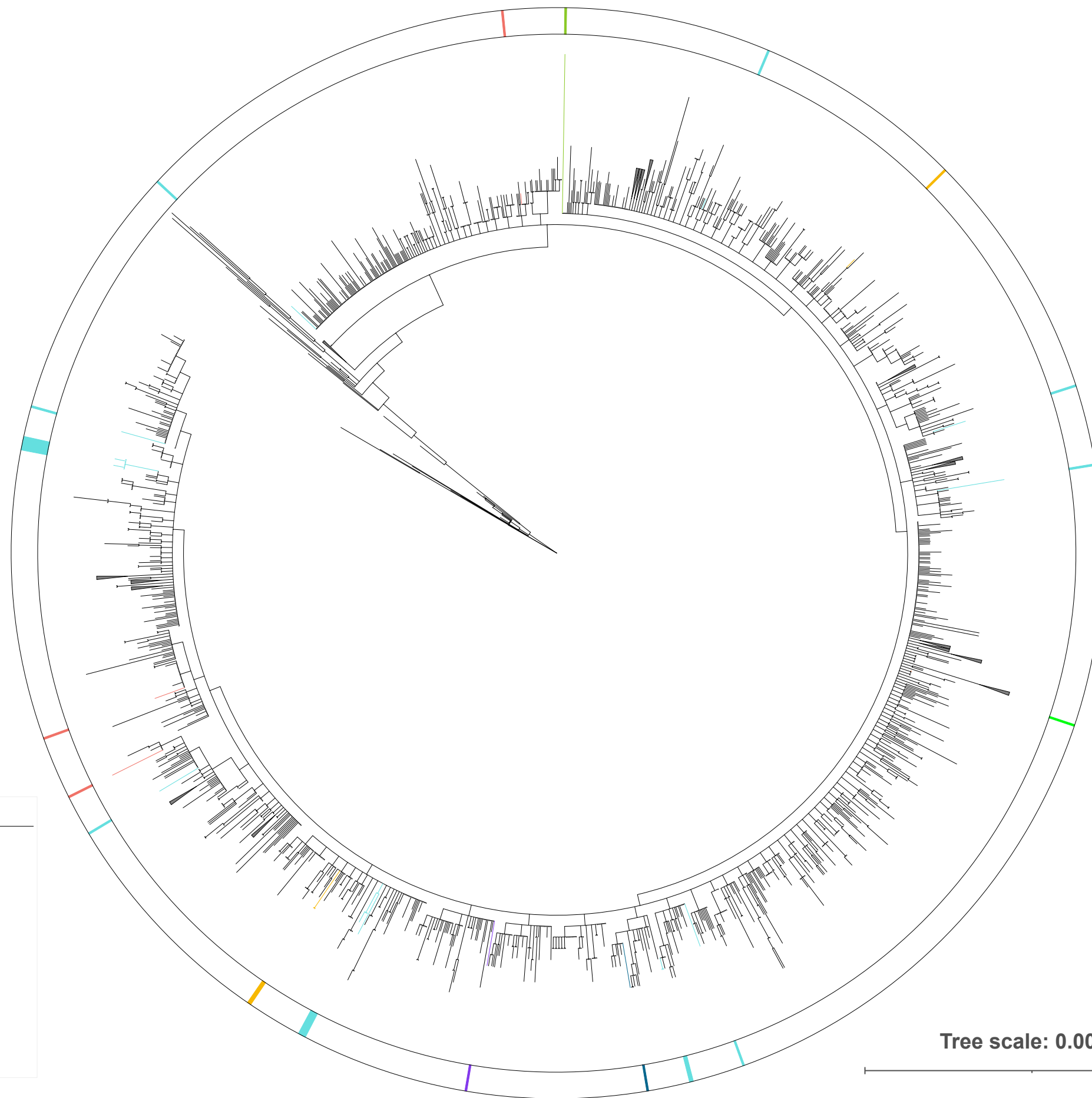

Tree scale: 0.001

Supplement: FIG S4 [file mbio.02315-21-sf004.pdf]
